# A novel EHD1/CD44/Hippo/SP1 positive feedback loop potentiates stemness and metastasis in lung adenocarcinoma

**DOI:** 10.1002/ctm2.836

**Published:** 2022-04-29

**Authors:** Yuechao Liu, Yang Song, Mengru Cao, Weina Fan, Yaowen Cui, Yimeng Cui, Yuning Zhan, Ruixue Gu, Fanglin Tian, Shuai Zhang, Li Cai, Ying Xing

**Affiliations:** ^1^ The Fourth Department of Medical Oncology Harbin Medical University Cancer Hospital Harbin China; ^2^ The First Department of Orthopedic Surgery The Second Affiliated Hospital of Harbin Medical University Harbin China

**Keywords:** EHD1, hippo signalling pathway, LUAD, metastasis, stemness

## Abstract

**Background:**

There is growing evidence that endocytosis plays a pivotal role in cancer metastasis. In this study, we first identified endocytic and metastasis‐associated genes (EMGs) and then investigated the biological functions and mechanisms of EMGs.

**Methods:**

Cancer stem cells (CSCs)‐like characteristics were evaluated by tumour limiting dilution assays, three‐dimensional (3D) spheroid cancer models. Microarray analysis was used to identify the pathways significantly regulated by mammalian Eps15 homology domain protein 1 (EHD1) knockdown. Mass spectrometry (MS) was performed to identify EHD1‐interacting proteins. The function of EHD1 as a regulator of cluster of differentiation 44 (CD44) endocytic recycling and lysosomal degradation was determined by CD44 biotinylation and recycling assays.

**Results:**

EHD1 was identified as a significant EMG. Knockdown of EHD1 suppressed CSCs‐like characteristics, epithelial–mesenchymal transition (EMT), migration and invasion of lung adenocarcinoma (LUAD) cells by increasing Hippo kinase cascade activation. Conversely, EHD1 overexpression inhibited the Hippo pathway to promote cancer stemness and metastasis. Notably, utilising MS analysis, the CD44 protein was identified as a potential binding partner of EHD1. Furthermore, EHD1 enhanced CD44 recycling and stability. Indeed, silencing of CD44 or disruption of the EHD1/CD44 interaction enhanced Hippo pathway activity and reduced CSCs‐like traits, EMT and metastasis. Interestingly, specificity protein 1 (SP1), a known downstream target gene of the Hippo‐TEA‐domain family members 1 (TEAD1) pathway, was found to directly bind to the *EHD1* promoter region and induce its expression. Among clinical specimens, the EHD1 expression level in LUAD tissues of metastatic patients was higher than that of non‐metastatic patients.

**Conclusions:**

Our findings emphasise that EHD1 might be a potent anti‐metastatic target and present a novel regulatory mechanism by which the EHD1/CD44/Hippo/SP1 positive feedback circuit plays pivotal roles in coupling modules of CSCs‐like properties and EMT in LUAD. Targeting this loop may serve as a remedy for patients with advanced metastatic LUAD.

## Graphical abstract

1. EHD1 interacts with the CD44‐ICD, promoting CD44 recycling and upregulating CD44 expression.
2. CD44 upregulation inhibits Hippo signalling activity, activating expression of downstream target genes involved in stemness and EMT.
3. SP1, as a downstream target of Hippo signalling, in turn transcriptionally activates EHD1 expression, finally forming an EHD1/CD44/Hippo/SP1 positive feedback loop that drives LUAD metastasis.

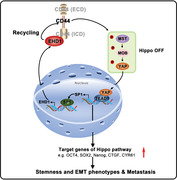

## 1 INTRODUCTION

Lung carcinoma is the second most often diagnosed cancer and the major cause of cancer mortality globally, with an anticipated 2 200 200 new cases and 1 800 000 deaths in 2020.^1^, ^2^ Over half of lung cancer patients exhibit evidence of local or distant metastasis at initial diagnosis, and the 5‐year relative survival rate of patients with advanced metastatic lung cancer is only 4%.^3^ Lung adenocarcinoma (LUAD) is the most predominant histological type of lung cancer (greater than 40% of cases), and its relative frequency is increasing.^4^, ^5^ Because of the tendency of LUAD to metastasise to lymph nodes, the contralateral lung and distant organs, its outcome remains unsatisfactory even after treatment with currently available therapies.^6^ Thus, efforts to better understand the potential oncogenic molecular mechanisms governing LUAD metastasis and to find effective therapeutic targets for metastatic LUAD are urgently needed.

Cancer invasion and metastasis involve a series of sophisticated cascade events.^7^, ^8^ It is essential that during metastasis, tumour cells with versatile self‐reprogramming abilities, must convert from an epithelial to a mesenchymal state and gain stemness properties to defend against attacks from hostile immune and apoptotic signals; these transformed cells can ultimately survive and contribute to local cancer recurrence.^9^, ^10^ Thus, it is well recognised that the acquisition of both cancer stem cells (CSCs)‐like properties and the epithelial–mesenchymal transition (EMT) of metastatic cancer cells are considered to be two fundamental events in the multistep process of cancer metastasis.^11^ Moreover, EMT and CSCs traits as a coupling key concept is considered a ‘dangerous dynamic duo’ in the tumour metastatic process.^12^ An active EMT program endows cancer cells with characteristics of stem cells and capabilities similar to those of CSCs.^13^ In turn, the stemness of cancer cells sustains the plasticity of the transition between the epithelial and mesenchymal states.^14^ Obviously, cancer cells that concurrently undergo EMT and possess CSCs‐like properties have potent metastatic potential.

Endocytosis is controlled by an elaborate network of endocytic genes.^15^ Eps15 homology domain protein 1 (EHD1) has been well characterised to regulate several specific steps in receptor‐mediated endocytic recycling.^16^ The receptors and their associated proteins undergo retrieval and recycling to the plasma membrane through the endocytic recycling route as a result of a binary choice, thereby avoiding the fate of lysosomal degradation.^17^ Therefore, EHD1 can affect or promote the signalling outputs of receptors.^18^, ^19^ The trafficking of a wide array of endocytosed receptors, including epidermal growth factor receptor (EGFR) and insulin‐like growth factor 1 receptor (IGF‐1R), is modulated by EHD1.^18^, ^19^, ^20^, ^21^, ^22^ EHD1 has been found to be a significantly effective inducer during cancer progression.^23^, ^24^, ^25^, ^26^, ^27^, ^28^, ^29^, ^30^, ^31^, ^32^, ^33^, ^34^, ^35^, ^36^ Although previous work has supported the roles of EHD1 in regulating metastasis,^35^, ^36^ the functions and mechanisms of EHD1 remain largely unknown.

In our current study, EHD1 was first postulated and then validated to be a key regulator of Hippo signalling activity and cluster of differentiation 44 (CD44) endocytic recycling. Moreover, we found that specificity protein 1 (SP1), a target gene of the Hippo pathway, can activate the transcription of EHD1, thus forming a positive feedback loop. Our experimental results indicate that the achieved EHD1/CD44/Hippo/SP1 feedback loop and concomitant Hippo signalling deactivation not only enhance CSCs‐like traits but also lead to EMT and metastasis of LUAD cells.

## 2 MATERIALS AND METHODS

### 2.1 Cell lines

The human LUAD cell lines A549 and H1299 were purchased from the American Type Culture Collection (Manassas, USA) and cultured in RPMI 1640 medium (Gibco, USA) supplemented with 10% foetal bovine serum (FBS, Gemini, USA) and 100 mg/ml penicillin/streptomycin/amphotericin B (SC120‐01, Sevenbio, Beijing, China). These two cell lines were applied in our study within forty passages.

### 2.2 Vectors, plasmids and stable cell lines

To knock down endogenous EHD1, three short hairpin RNA (shRNA) oligonucleotides were separately cloned to construct the hU6‐MCS‐CMV‐Puromycin‐EHD1 plasmid. Silencing of CD44 and SP1 was implemented with commercial shRNAs targeting CD44 and SP1. Lentiviral particles containing these shRNAs were purchased from GeneChem (Shanghai, China). The detailed sequences are shown in Table S1.

The full‐length and truncated sequences of EHD1 were inserted into the pcDNA3.1‐flag vector at the BamHI (GGATCC) and EcoRI (GAATTC) cloning sites to construct EHD1 full‐length [EHD1‐FL, amino acids (aa) 1–534)], C‐terminal EH domain deletion (EHD1‐∆EH, aa 1–439), P‐loop domain (EHD1‐P, aa 1–190) and EH domain (EHD1‐EH, aa 439–534) expression plasmids, as described previously.^37^ Moreover, truncated sequences of CD44 were inserted into the pcDNA3.1‐Myc vector at the BamHI (GGATCC) and EcoRI (GAATTC) cloning sites to construct the CD44 full‐length (CD44‐FL, aa 1–742), C‐terminal intracellular domain deletion (CD44‐ΔICD, aa 1–671), extracellular domain (CD44‐ECD, aa 1–649) and intracellular domain (CD44‐ICD, aa 671–742) expression plasmids.^38^ All plasmids were constructed by General Biosystems (Anhui, China).

Stable EHD1 shRNA‐expressing (shEHD1‐1 and shEHD1‐2) clones and control shRNA‐expressing [shCtrl, negative control (NC)] clones were established according to the manufacturer's protocol. EHD1‐FL [wild‐type (WT) *EHD1*], EHD1‐∆EH (mutant *EHD1*) and control lentiviral vectors were transduced into shEHD1‐1 (EHD1^KD^) cells to establish stable clones (EHD1^KD+WT^, EHD1^KD+MUT^ and EHD1^KD+Ctrl^, respectively). All stable cell lines were subjected to GFP sorting and treatment with 1 μg/ml puromycin.

### 2.3 Three‐dimensional (3D) spheroid cancer models

Two spheroid cancer models were established according to previous studies.^39^ The first model was established using semisolid medium. Growth factor‐reduced Matrigel^®^ Basement Membrane Matrix (300 μl per well, Corning, NY) was diluted in 300 μl of DMEM/F12 (Gibco, USA) supplemented with 20 ng/ml EGF (Gibco, USA), 10 ng/ml bFGF (Gibco, USA), B27 (1:50, Thermo Fisher Scientific, USA), N2 (1:100, Thermo Fisher Scientific, USA) and ITS (1:100, Sigma, USA) in a 24‐well plate (Corning, USA) and was then incubated at 37°C for 1 h. Then, 300 μl of the cell suspensions (1×10^5^ cells/ml) were inoculated into the wells and the cells were cultured for 10 days. Count spheres larger than 100 μm in diameter.

The second spheroid cancer model was generated with stem cell medium. Cell suspensions (300 μl per well, 1×10^5^ cells/ml) were stated in DMEM/F12 (supplemented with EGF, bFGF, B27, N2 and ITS) and seeded into 6‐well ultralow attachment plates (Corning, USA). After 14 days, tumour spheres with a diameter greater than 50 μm were counted.

### 2.4 Holoclone assays

The method for holoclone assays was adapted from a previously reported study.^40^ Five hundred cells were inoculated into each well of a six‐well plate. After 10–14 days, secondary colonies were subdivided into holoclones, meroclones or paraclones according to their morphological characteristics.^41^ The secondary colonies were counted. One hundred colonies were randomly selected in each assay, and the assay was repeated three independent times.

### 2.5 Flow cytometric analysis of CD44‐ and CD133‐positive cells

The expression of CD44 and CD133 which was the cell surface markers was examined using flow cytometry as described previously.^42^ Cell suspensions (1 ml, 1×10^6^ cells/ml) were centrifuged at 2000 rpm at 4°C for 2 min. Then, 50 μl of PBS was added to the cells and thoroughly mixed by pipetting. Anti‐CD44‐FITC (Abcam, ab27285) and anti‐CD133‐PE (BioLegend, Cat# 393904) antibodies were added to LUAD cells and the cells were incubated on a shaker at 4℃ keeping away from light for 30 min. After washing, the LUAD cells were fixed with 1% paraformaldehyde. Flow cytometry was conducted in a FACSCalibur (BD Biosciences, San Jose, CA, USA), and FlowJo software was used to analyse the data.

### 2.6 Microarray profiling

Briefly, total RNA for microarray profiling was obtained from H1299‐derived cells (NC and EHD1^KD^). An Affymetrix GeneChip PrimeView Human Gene Expression Array was adapted for microarray profiling to identify gene expression profiles following the manufacturer's instructions. ‘Canonical pathway analysis’ in Ingenuity Pathway Analysis (IPA) software (version 2018; Ingenuity Systems; QIAGEN) was used to determine the enrichment of differentially expressed genes in the canonical signalling pathways.^43^

### 2.7 Subcellular fractionation

Cell fractionation was carried out using a Minute^TM^ Cytoplasmic and Nuclear Extraction Kit (Invent Biotechnologies. Inc., USA) according to the manufacturer's instructions and previous reports.^44^

### 2.8 Dual‐luciferase reporter assay

A549 and H1299 cells were grown to approximately 80% confluence and were then transfected with the pGL3‐Basic and YAP luciferase plasmids (Hanyin Biotechnology Limited Company, Shanghai, China). After 48 h, a luciferase reporter assay system was used to evaluate the transcriptional level of YAP. The relative luciferase activity (firefly luciferase/Renilla luciferase) in all groups was calculated to represent Hippo signalling activity.

### 2.9 Mass spectrometry (MS)

To identify the potential interacting proteins of EHD1, MS analysis was performed. A549 cells were lysed and immunoprecipitated with IgG or an anti‐EHD1 antibody. The bound proteins were eluted by SDS‐PAGE. Based on the identified amino acid sequences, EHD1‐interacting client proteins were identified in UniProt (https://www.uniprot.org/uniprot/).

### 2.10 Ubiquitination assay

The indicated LUAD cells were co‐transfected with Lys48 (K48)‐ or Lys63 (K63)‐linked haemagglutinin‐Ubiquitin or total haemagglutinin‐Ubiquitin. Cells were treated with a lysosome inhibitor (NH_4_Cl, Aladdin, China) to increase the level of ubiquitinated CD44. Immunoprecipitation (IP) assays were conducted to obtain ubiquitinated CD44 immunoprecipitated by an anti‐haemagglutinin antibody or IgG.

### 2.11 Flow cytometric recycling assay

A cell surface receptor CD44 recycling assay was conducted by flow cytometry as detailed elsewhere.^45^, ^46^ In brief, cells were grown to 80% confluence and were then starved overnight. In our study, four sample groups were prepared. One sample group was considered as the *T*_0_ group. The other three sample groups were treated with hyaluronic acid (HA, 0.3 mg/ml, MCE, USA) for 1 h at 4°C, and one of these groups was considered as the pulse group. The remaining two groups were incubated with HA for 15 min at 37°C to induce CD44 internalisation. Next, an acid/salt solution (0.2 M acetic acid and 0.5 M NaCl) was used to remove bound HA. The two groups of cells were chased for 1 or 2 h at 37°C to allow CD44 recycling before fixation. Cell surface CD44 was labelled using an anti‐CD44‐FITC antibody (Abcam, ab27285). The mean fluorescence intensities (MFIs) were analysed by flow cytometry. The surface levels of CD44 at 1 and 2 h were defined as MFI(t). Furthermore, the relative surface levels of CD44 at each time point were calculated using the formula [MFI(*t*) – MFI(pulse)/MFI(*T*_0_) – MFI(pulse)] × 100.

### 2.12 Biotinylation and recycling assay

To examine the level of CD44 recycling, EZ‐Link Sulfo‐NHS‐SS‐Biotin (0.2 mg/ml, Thermo, Wilmington, DE, USA) was used to label CD44. The experimental procedure for this assay was described previously.^47^ In brief, cells were surface‐labelled with biotin at 4°C for 1 h. Next, the cells were treated with HA for 15 min at 37°C to induce the internalisation of CD44. The cells were then washed three times (at 4°C for 10 min each) with MesNa solution (20 mM, Aladdin, China) to remove the remaining biotin. Then, 1/4 of the cells were retained and further evaluated to determine the total biotinylated CD44 level. The rest of the cells were split into three equal portions to examine the recycling of internalised CD44 for 0.5, 1.0 and 2.0 h by rewarming at 37°C in medium without serum. MesNa solution was employed again to remove the biotin conjugated to CD44 at the cell surface. The total and remaining biotinylated CD44 were quantified by enzyme‐linked immunosorbent assay (ELISA) using microtitre wells coated with an anti‐CD44 antibody (Jianglai Biotechnology Company, 1:500). The recycling percentage (recycling%) was calculated with the following formula: recycling% = (total − remaining)/total × 100%.

### 2.13 Animal models

The BALB/c nude mouse experiments conducted in this study used 6‐week‐old female nude mice. The operations were conducted in accordance with the Guide for the Care Use of Laboratory Animals of the Harbin Medical University Institutional Animal Care and Use Committee.

To establish the metastasis model in nude mice, 42 nude mice (Beijing Vitalstar Biotechnology, China) were randomly divided into seven groups: NC, EHD1^KD^, EHD1^KD+Ctrl^, EHD1^KD + WT^, EHD1^KD + MUT^, EHD1^KD + WT^ + DMSO and EHD1^KD + WT^ + verteporfin (VP) groups. The indicated LUAD cells in the logarithmic growth phase were digested by a conventional trypsinisation and centrifugation method. Each type of cell as indicated (1×10^6^ cells per mouse) was separately injected intravenously (i.v.) into mice on day zero. VP (100 mg/kg, three times weekly for three consecutive weeks) or DMSO was injected intraperitoneally 10 days after tail vein injection of cells. After routine breeding and observation for 70 days, the nude mice were sacrificed. The number of lung metastatic foci was determined and recorded. The metastatic tissues were harvested and fixed in paraformaldehyde for the subsequent experiment.

The *in vivo* limiting dilution assay was carried out according to previous reports.^48^, ^49^ In brief, 2×10^4^, 1×10^4^ and 5×10^3^ indicated cells were subcutaneously inoculated into mice (*n* =  6 mice per group). The stem cell frequency was calculated with ELDA software (http://bioinf.wehi.edu.au/software/elda/).

### 2.14 Clinical specimens

The ethical approval was obtained from the Ethics Review Committee at Harbin Medical University Cancer Hospital. A total of 113 clinical tissue samples, which were fixed with formalin and embedded in paraffin, were acquired form LUAD patients (58 patients with lymph node metastasis, 55 patients without lymph node metastasis). All the patients underwent surgical resection of tumours between January 2010 and August 2016 at Harbin Medical University Cancer Hospital.

### 2.15 Statistical analysis

A detailed description of the statistical analysis is presented in the Supplementary Information.

## 3 RESULTS

### 3.1 Identification of endocytic and metastasis‐associated genes (EMGs) in LUAD

**FIGURE 1:**
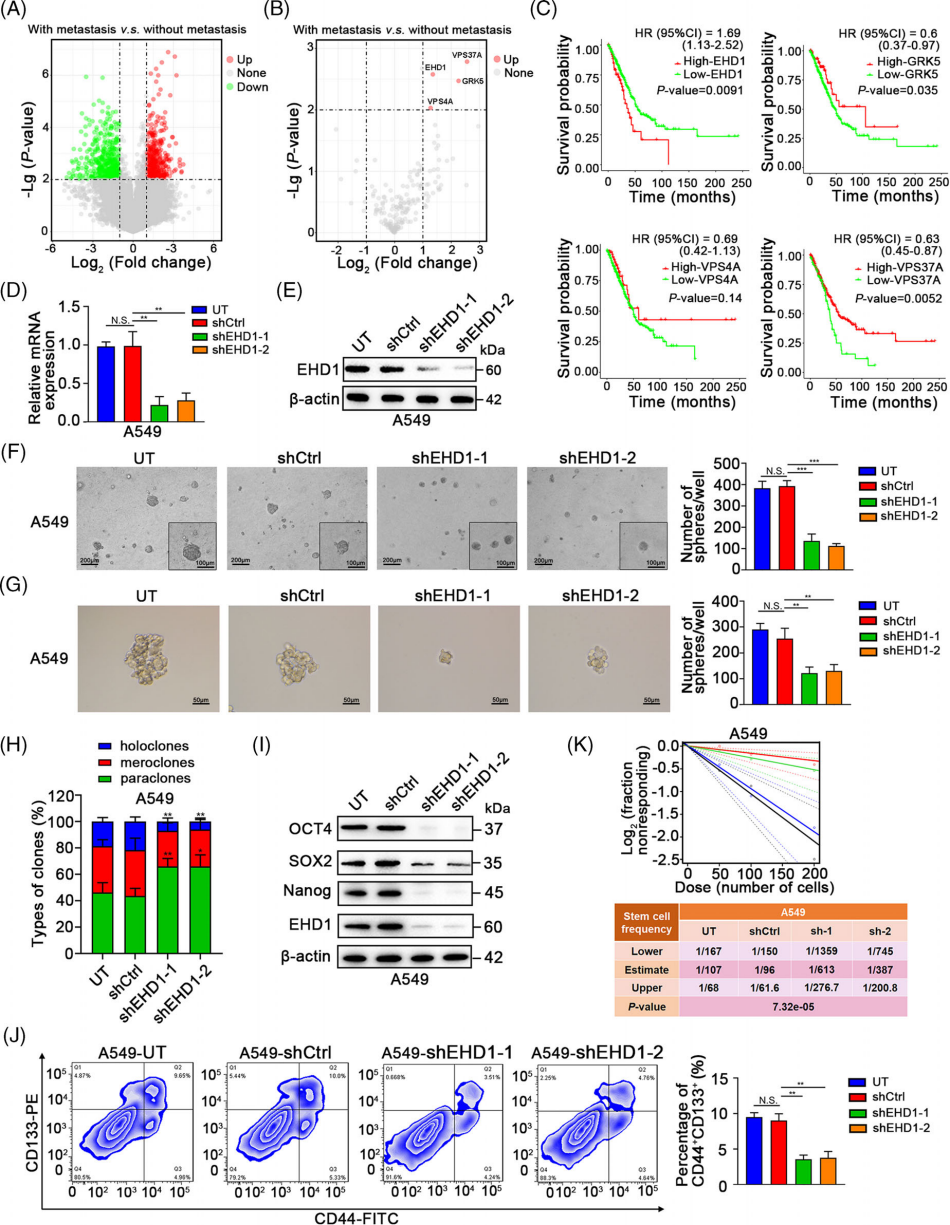
Identification of EMGs in LUAD. (A) A volcano plot indicating upregulated (red dots) and downregulated (green dots) genes in the LUAD tissues of patients with bone metastasis compared with those without bone metastasis. Black dotted lines represent a cut‐off range of 2.0‐fold and *p* < .01. (B) A volcano plot showing 4 upregulated (red dots) genes associated with endocytosis in the LUAD tissues of the patients with bone metastasis compared with those without bone metastasis. Black dotted lines represent a cut‐off range of 2.0‐fold and *p* < .01. *VPS37A*, *EHD1*, *GRK5* and *VPS4A* were identified as the candidate EMGs. (C) Kaplan–Meier curves for the overall survival of patients with LUAD according to the expression levels of the candidate EMGs. HR, hazard ratio; CI, confidence interval. (D and E) The effects of EHD1 knockdown in A549 cell lines were examined by qRT‐PCR and Western blot analysis. (F–H) The effect of EHD1 depletion on the stemness of A549 cells was determined *in vitro*. (F) 3D spheroid assays using semisolid medium, (G) 3D spheroid assays using serum‐free medium and (H) holoclone assays showed the CSCs‐like traits of A549 cells. (I) The expression of stemness‐related markers in A549 cells. (J) Flow cytometric analysis of CD133‐ and CD44‐positive cells. (K) In the *in vitro* limiting dilution assays, the numbers of wells in 96‐well plates that contained tumour spheres were determined (upper panel). The stemness of A549 cells with or without EHD1 knockdown was estimated as the stem cell frequency (bottom panel). The data are shown as the mean ± standard deviation (SD) values. *p* > .05 was considered not significant (N.S.), *p < .05, **p < .01 and ***p < .001

Compared to the expression levels in LUAD tissues of the patients without bone metastasis, 873 genes were differentially expressed in those with bone metastasis based on GEO (GSE175601) database (Figure 1A). Totally, 181 endocytic genes were obtained from *Molecular Signatures Database v7.5* (http://www.gsea‐msigdb.org/gsea/msigdb/geneset_page.jsp?geneSetName = KEGG_ENDOCYTOSIS&keywords = Endocytosis). Four genes, *VPS37A*, *EHD1*, *GRK5* and *VPS4A*, were found to be the candidate EMGs (Figure 1B and Table S2). Furthermore, Kaplan–Meier plots showed that only high EHD1 expression was consistently an unfavourable predictor for LUAD (Figure 1C). Because EHD1 was upregulated in LUAD tissues of patients with distant‐metastasis and was a poor prognostic factor, it was identified as a significant EMG and chosen for further analyses.

### 3.2 EHD1 promotes LUAD cell stemness, EMT and metastasis both *in vitro* and *in vivo*

CSCs‐like characteristics and EMT are two paramount attributes that drive the metastatic cascade. To provide comprehensive data and reliable evidence for the functional role of EHD1 in CSCs‐like properties, *EHD1* was knocked down by two independent EHD1 shRNAs in A549 and H1299 cells. Then, we generated stable EHD1 shRNA‐expressing clones (shEHD1‐1 and shEHD1‐2) and a shCtrl clone. Successful knockdown of EHD1 was validated by quantitative reverse transcription‐PCR (qRT‐PCR) and Western blotting (Figure 1D and E and Figure S1A and B). In the 3D spheroid cancer models, fewer spheres were formed in the EHD1‐knockdown groups than in the corresponding shCtrl or untreated (UT) groups (Figure 1F and G and Figure S1C and D). Among the holo‐, mero‐ and paraclones, the holoclones had the greatest reproductive ability and were characterised by a stem‐like phenotype.^40^, ^41^ Knockdown of EHD1 significantly decreased the number of holoclones (Figure 1H and Figure S1E).

Consistent with the above findings, the levels of the stem cell markers OCT4, SOX2 and Nanog were decreased by EHD1 depletion, as determined by Western blotting (Figure 1I and Figure S1F). Moreover, flow cytometric analysis of surface markers (CD44 and CD133) indicated that the number of CD133‐ and CD44‐positive cells was also decreased after EHD1 knockdown (Figure 1J). In addition, the limiting dilution assay demonstrated that targeting EHD1 with shRNAs decreased the self‐renewal capacity of LUAD cells (Figure 1K and Figure S1G). To investigate whether EHD1 expression can directly affect CSCs‐like traits, EHD1 expression was restored in shEHD1‐1 cells (EHD1^KD^). Restoration of WT EHD1 expression in A549 and H1299 cells (EHD1^KD+WT^) compared with the corresponding control cells (EHD1^KD+Ctrl^), was verified using qRT‐PCR and Western blotting (Figure S2A and B). As expected, overexpression EHD1 enhanced the CSCs properties of LUAD cells, as further indicated by the 3D spheroid cancer models (Figure S2C and D), the holoclone assay (Figure S2E), stem cell marker evaluation (Figure S2F), flow cytometric analysis of CD133‐ and CD44‐positive cells (Figure S2G), and limiting dilution assay (Figure S2H). Similar results of EHD1 restoration were observed in H1299 cells (Figure S3). Our results suggest that EHD1 is essential for the stemness of LUAD cells.

**FIGURE 2:**
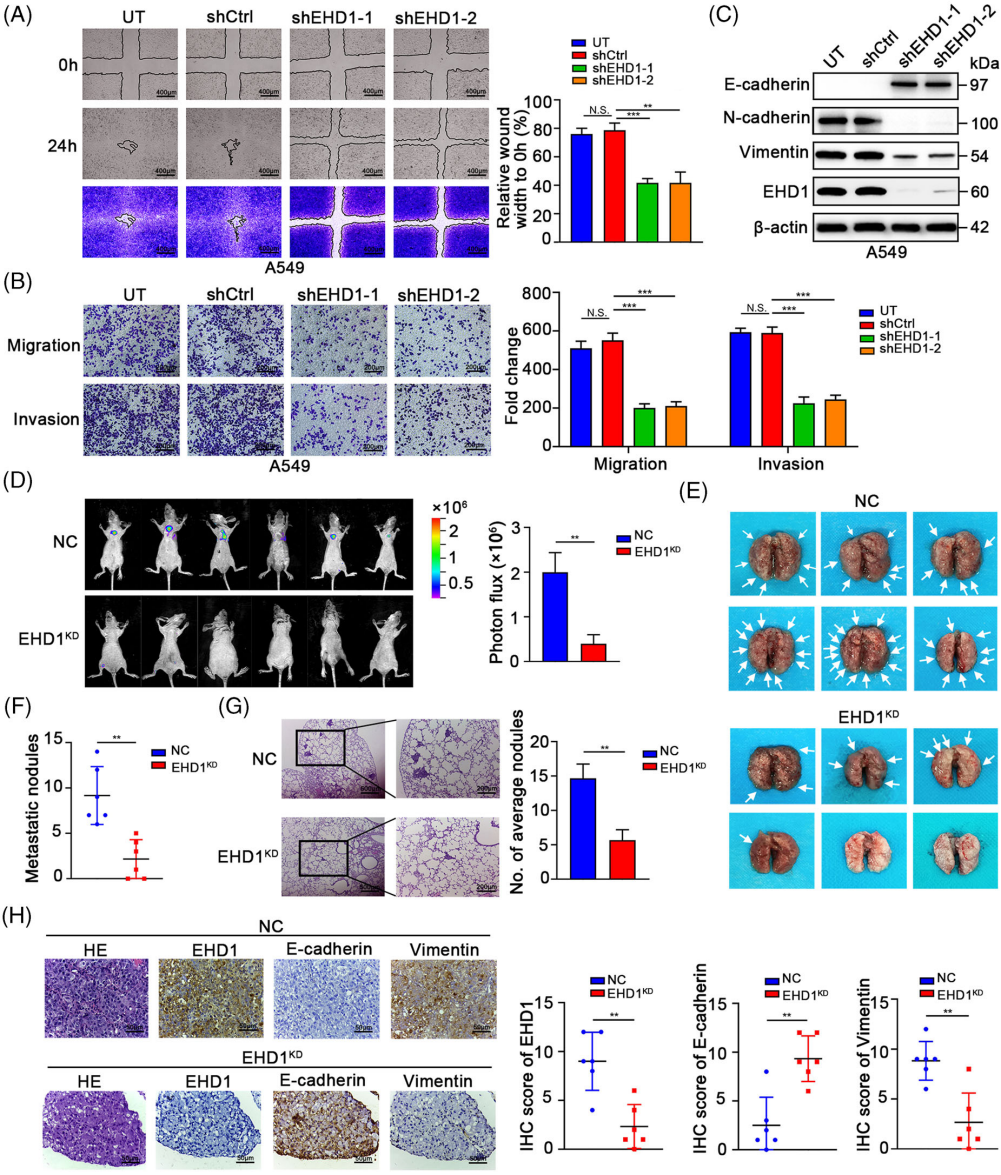
Knockdown of EHD1 inhibits the metastasis of LUAD cells. (A) Representative image of the wound healing assay showing the effect of EHD1 knockdown on the migration ability and motility of A549 cells. The quantitative and statistical analyses based on the area of the wound indicated the cell migration ability and cell motility. (B) The Transwell assay showing the effects of EHD1 knockdown on the migration and invasion of A549 cells. (C) The expression of EMT‐related markers in A549 cells after EHD1 knockdown. (D) The images show the bioluminescence signal intensities in each BALB/c nude mouse on day 70 after tail vein injection of NC or EHD1^KD^ cells (left panel). The bar charts show the results of quantitative and statistical analyses of the bioluminescence signal intensities in the NC and EHD1^KD^ groups (right panel). (E) Pulmonary metastases in nude mice after tail vein injection of NC or EHD1^KD^. The white arrows indicate lung metastatic foci. (F) Number of metastatic nodules in the lungs of nude mice in the NC and EHD1^KD^ groups. (G) Representative H&E staining images of lung tissues from nude mice injected with NC or EHD1^KD^ (left panel). The numbers of spontaneous lung metastatic nodules in mice were quantified (right panel). (H) Representative images of H&E staining and IHC staining for EHD1 and EMT‐related proteins in metastatic lung tissues formed by NC or EHD1^KD^ (left panel). The bar charts show the results of quantitative and statistical analyses of IHC assays (right panel). The data are shown as the mean ± SD values. *p* > .05 was considered N.S., **p < .01 and ***p < .001

The wound healing and Transwell assays indicated that EHD1 knockdown inhibited the migration, invasion and motility of A549 cells (Figure 2A and B) and H1299 cells (Figure S4A and B). Knockdown of EHD1 resulted in morphological changes in LUAD cells from a mesenchymal spindle‐like morphology to an epithelial cuboidal‐shaped phenotype (Figure S4C and D). Western blot analysis showed increased expression of epithelial markers such as E‐cadherin and decreased expression of mesenchymal markers such as N‐cadherin in the EHD1‐knockdown groups compared with the corresponding shCtrl or UT groups (Figure 2C and Figure S4E). In the *in vivo* metastasis assays, knockdown of EHD1 repressed the lung metastatic capability of LUAD cells in tail vein‐injected nude mice, as indicated by the lower luciferase signals (Figure 2D) and lower numbers of metastatic foci in the lungs (Figure 2E–G) compared with those in NC mice injected with shCtrl cells. Subsequently, haematoxylin and eosin (H&E) staining and immunohistochemistry (IHC) were performed on serial sections of lung tissues from tail vein‐injected mice. Less Vimentin but more E‐cadherin staining was observed in lung metastasis tissues from EHD1^KD^ group than in those from NC group (Figure 2H). Conversely, EHD1 overexpression strongly promoted invasion and metastasis and restored the EMT program in A549 and H1299 cells in which re‐exhibited a less polarised and spindle‐like morphology (Figure S5). Our comprehensive experimental data indicate that EHD1 enables to operate the coupling modules of ‘dangerous dynamic duo’, namely, CSCs‐like properties and EMT, leading to LUAD metastasis.

### 3.3 EHD1 suppresses Hippo signalling activation

**FIGURE 3:**
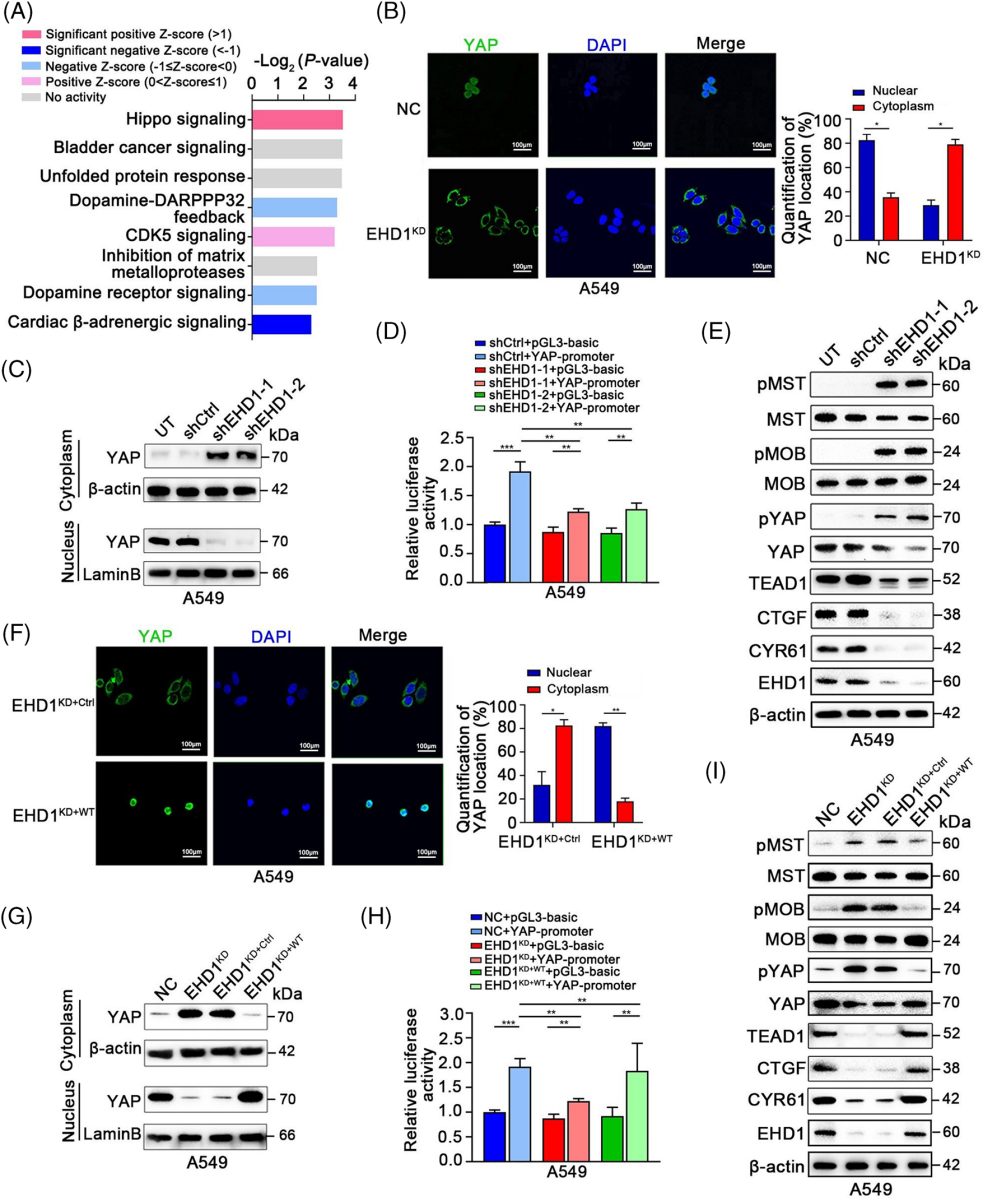
EHD1 inhibits Hippo signalling activation. (A) ‘Canonical pathway analysis’ in IPA software was used to summarise the enrichment of differentially expressed genes between NC and EHD1^KD^. All signalling pathways were ranked by Log_2_ (*p* value). (B) Representative IF images showing the localisation of YAP in NC and EHD1^KD^ (left panel). Green indicates YAP IF staining; blue indicates DAPI staining. The bar graphs show the statistical analysis of YAP localisation in A549 cells (right panel). (C) The expression levels of YAP in the cytoplasm and nucleus were determined by subcellular fractionation assays in NC and EHD1^KD^. β‐actin was used as the control for cytoplasmic expression, while Lamin B was used as the control for nuclear expression. (D) Luciferase reporter assays showing YAP transcriptional activity in NC and EHD1^KD^ transfected with the empty vector or YAP plasmid. (E) Protein expression levels of core Hippo signalling pathway components in A549 cells. (F) Representative IF images validating the localisation of YAP in A549 cells with or without EHD1 overexpression (left). Bar graphs showing the statistical analysis of YAP localisation in A549 cells (right). (G) The expression levels of YAP in the cytoplasm and nucleus of A549 cells with or without EHD1 restoration were determined by subcellular fractionation assays. β‐actin was used as the control for cytoplasmic expression, while Lamin B was used as the control for nuclear expression. (H) A luciferase reporter assay was performed to determine YAP transcriptional activity in A549 cells with or without EHD1 restoration that were transfected with the empty vector or YAP plasmid. (I) The expression of core components and downstream targets of the Hippo signalling pathway was detected by Western blotting. The data are shown as the mean ± SD values. *p < .05, **p < .01 and ***p < .001

To elucidate the mechanism by which deregulation of EHD1 contributes to LUAD metastasis, we conducted global gene expression microarray profiling in NC and EHD1^KD^. ‘Canonical pathway analysis’ in IPA software indicated that based on the differentially expressed genes (*p* < .05 and absolute fold change > 1.3), Hippo signalling pathway was notable and was the highest‐ranked pathway according to the *p* value (Figure 3A). Thus, we performed immunofluorescence (IF) and subcellular fractionation assays to analyse YAP subcellular localisation, which indicates Hippo signalling activity.^50^ As shown in Figure 3B and C, more YAP was retained in the cytoplasm in the EHD1‐knockdown groups than in the corresponding shCtrl or UT groups, suggesting that EHD1 inhibits the activation of Hippo signalling (Figure 3B and C and Figure S6A and B). Consistent with these findings, YAP luciferase reporter activity was suppressed in LUAD cells after knockdown of EHD1 (Figure 3D and Figure S6C). Moreover, after EHD1 depletion, the phosphorylation levels of the core Hippo signalling pathway components (MST, MOB and YAP) were increased, whereas the expression level of TEAD1 (a key DNA‐binding platform for YAP) and the expression levels of CTGF and CYR61 (downstream transcriptional targets of Hippo signalling) were decreased (Figure 3E and Figure S6D). In contrast, EHD1 restoration inhibited Hippo signalling pathway activity in both A549 (Figure 3F–I) and H1299 cells (Figure S6E–H).

### 3.4 Hippo signalling is essential for EHD1‐mediated CSCs‐like traits and metastasis *in vitro* and *in vivo*

**FIGURE 4:**
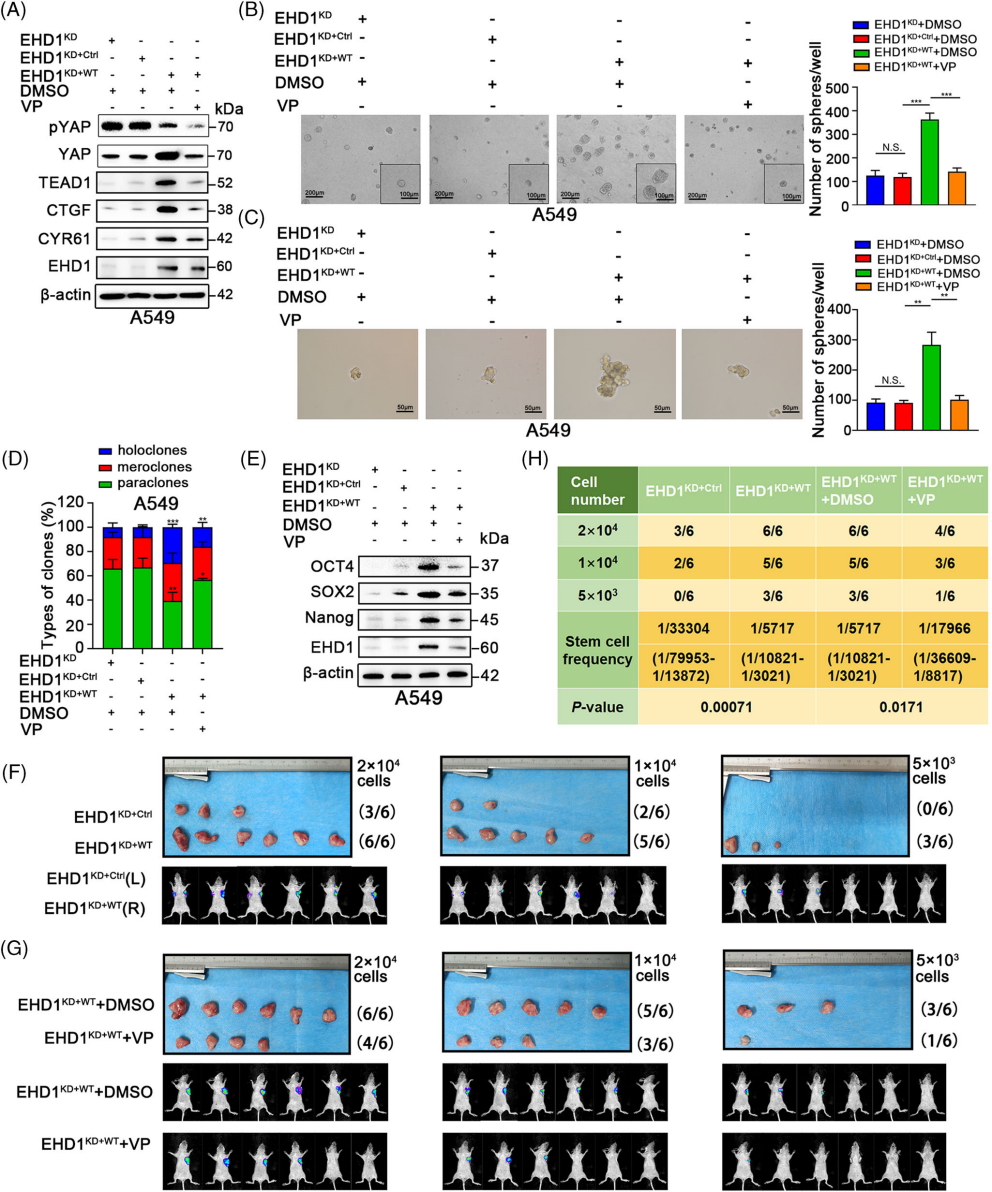
Hippo signalling is essential for EHD1‐mediated enhancement of stemness *in vitro* and *in vivo*. (A) Western blot analysis validated the effect of treatment with VP (1 μM) or DMSO for 24 h on the Hippo signalling pathway in the EHD1^KD+WT^ group. The expression of core components and downstream targets of the Hippo signalling pathway was detected. (B‐C) The 3D spheroid cancer models detected the effect of VP treatment on stemness in the EHD1^KD+WT^ group. The bar graphs show the quantification of the number of spheres per well formed by A549‐derived cells. (D) The stemness of A549 cells treated with DMSO or VP was evaluated by clonal heterogeneity analysis. (E) Expression of stemness‐related proteins in A549‐derived cells treated with DMSO or VP. (F‐H) Hippo signalling is critical for the EHD1‐mediated enhancement of A549 cell stemness *in vivo*. (F) Photographs showing the efficiency of tumour generation or tumoursphere formation in nude mice (upper panels). The panels below the images show the bioluminescence signal intensities in nude mice injected subcutaneously with EHD1^KD+Ctrl^ (left) or EHD1^KD+WT^ (right). (G) Images showing the efficiency of tumour generation or tumoursphere formation in nude mice treated with DMSO or VP (upper panels). Bioluminescence signal intensities in nude mice injected subcutaneously with EHD1^KD+WT^ and treated with DMSO or VP are shown (lower panels). (H) The stemness of A549 cells was estimated as the stem cell frequency, with upper and lower 95% confidence intervals. The tumour engraftment efficiency *in vivo* was successfully reduced by VP treatment. The data are shown as the mean ± SD values. *p* > .05 was considered N.S., *p < .05, **p < .01 and ***p < .001

Hippo signalling plays critical roles in the stem cell phenotype, EMT and metastasis.^51^ To investigate whether EHD1 mediates the acquisition of malignant phenotypes in a Hippo signalling‐dependent manner, VP was used as a pharmacological tool to disrupt the TEAD‐YAP complex and suppress YAP1 transcriptional activity.^52^ The levels of p‐YAP, YAP, TEAD1, CTGF and CYR61 in A549 and H1299 cells treated with VP were lower than those in the EHD1^KD+WT^ group, suggesting that VP successfully functioned (Figure 4A and Figure S7A). VP significantly blocked the promoting effect of EHD1 on CSCs‐like traits, EMT, migration and invasion in both A549 cells (Figure 4B–E and Figure S8) and H1299 cells (Figure S7B–H). Using the *in vivo* limiting dilution assay, we found that EHD1 overexpression enhanced the efficiency of tumour generation or tumoursphere formation and enlarged the final xenograft size in nude mice (Figure 4F), whereas treatment with VP decreased the EHD1‐induced enhancement of tumoursphere formation efficiency and reversed cancer cell stemness (Figure 4G). The stem cell frequencies *in vivo* were calculated with ELDA software, and the results showed that EHD1 plays a promoting role in stemness; however, this effect was impaired by VP (Figure 4H). These data indicated that EHD1 regulates LUAD metastasis through the Hippo‐YAP signalling pathway.

### 3.5 EHD1 interacts with CD44 and facilitates its recycling, thus preventing its lysosomal degradation

**FIGURE 5:**
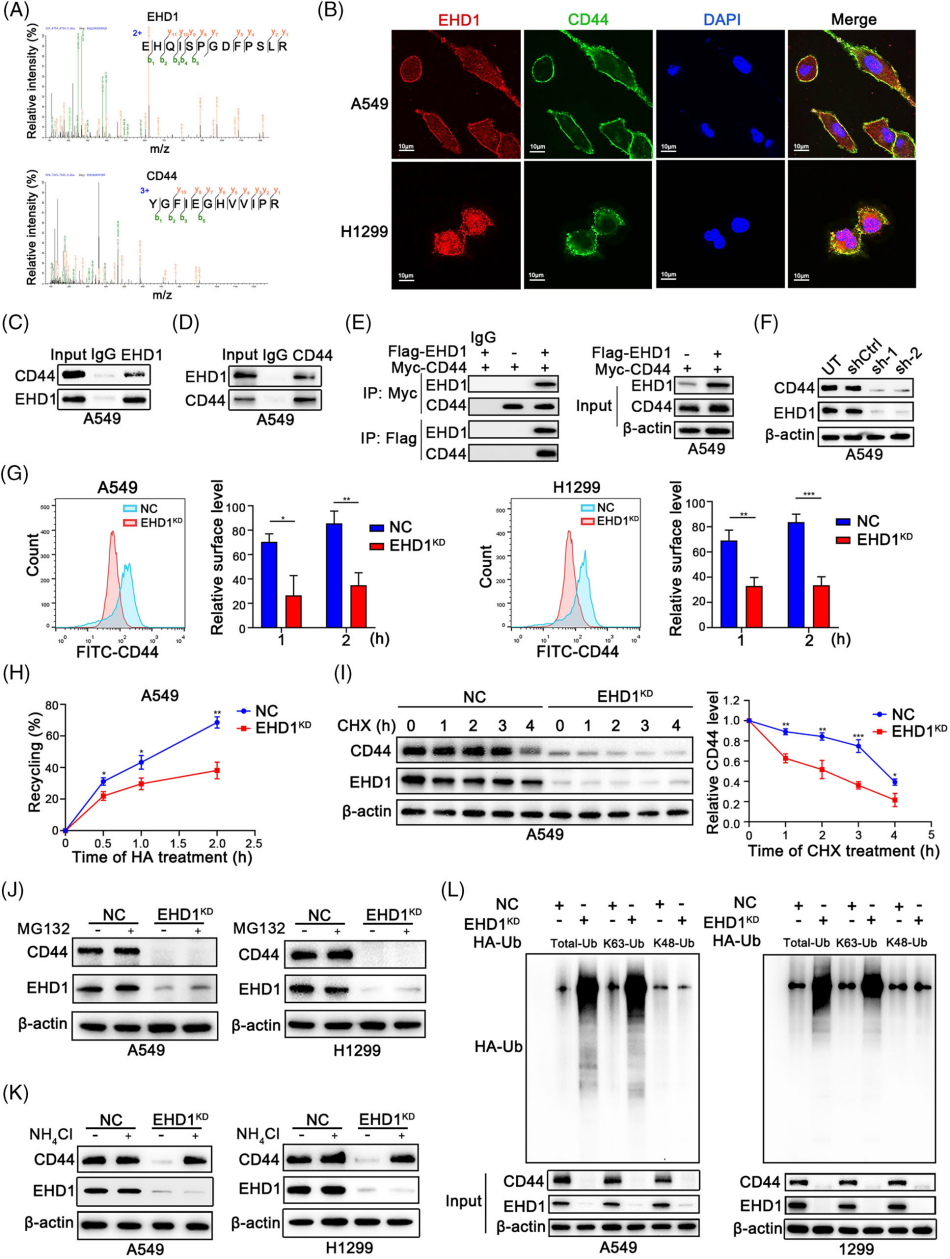
EHD1 interacts with CD44 and enhances its recycling, thus preventing its lysosomal degradation. (A) Amino acid sequences of peptides specifically associated with the EHD1 protein (top) or CD44 protein (bottom) were identified using MS. (B) IF staining was used to detect the colocalisation of EHD1 and CD44 in LUAD cells. Endogenous (C and D) and exogenous (E) IP experiments validated the interaction of EHD1 and CD44. (F) Western blot analysis was performed to examine the effect of EHD1 knockdown on the regulation of CD44 protein expression. (G) The MFI of relative surface CD44 was determined by FACS. Representative flow cytometry data and statistical analysis of cell surface CD44 in A549 (left) and H1299 cells (right) are shown. The relative surface level is related to the amount of the receptor that undergoes ligand fixation, stimulation, internalisation and recycling to the cell surface. (H) The biotinylation and recycling assay of CD44 by ELISA showed CD44 recycling in NC and EHD1^KD^ A549 cells. (I) A CHX chase assay was performed to analyse the half‐life of the CD44 protein in NC and EHD1^KD^ A549 cells. Cells were incubated in the presence of CHX (20 μg/ml) for 0, 1, 2, 3 or 4 h. (J) Western blot analysis of CD44 expression in NC and EHD1^KD^ treated with the proteasome inhibitor MG132 for 8 h. (K) Western blot analysis of CD44 expression in NC and EHD1^KD^ cells treated with NH_4_Cl, an inhibitor of the lysosomal pathway, for 8 h. (L) CD44 ubiquitination assays in LUAD cells transfected with the Total‐Ub, K63‐Ub or K48‐Ub plasmid after treatment with NH_4_Cl for 8 h. The data are shown as the mean ± SD values. *p < .05, **p < .01 and ***p < .001

To elucidate the mechanism by which EHD1 orchestrates Hippo cascade‐mediated CSCs‐like traits and metastasis, MS analysis of peptides and proteins was carried out to identify the proteins that interact with EHD1. Proteomic profiling of A549 cells identified 87 EHD1‐interacting client proteins, as well as the bait protein, EHD1 (Figure S9A and B). Surprisingly but encouragingly, the amino acid sequences of the peptides specifically belonging to CD44 protein (Figure 5A), a well‐known suppressor of Hippo signalling activation.^53^, ^54^ It has been reported that CD44 promotes phosphorylation of Merlin, leading to attenuated activation of the Hippo signalling pathway.^53^ EHD1 displayed similar effects to those of CD44 that EHD1 knockdown resulted in a decrease in the phosphorylation of Merlin in LUAD cells (Figure S9C). Through IF staining, co‐localisation assays and confocal imaging, we observed the co‐localisation of EHD1 and CD44 in both A549 and H1299 cells (Figure 5B). Furthermore, the interaction of EHD1 and CD44 was validated by IP. We detected bound CD44 after IP of EHD1 (Figure 5C and Figure S9D) and bound EHD1 after IP of CD44 (Figure 5D and Figure S9E). In LUAD cells co‐expressing Flag‐EHD1 and Myc‐CD44, exogenous EHD1 bound to CD44, and exogenous CD44 bound to EHD1 (Figure 5E and Figure S9F).

This physical interaction of EHD1 and CD44 indicated that EHD1 might regulate CD44. As determined by qRT‐PCR and Western blotting, the protein level of CD44 was decreased after EHD1 knockdown (Figure 5F and Figure S9H), but the CD44 mRNA level was unchanged (Figure S9G), suggesting that EHD1 acts as a posttranscriptional regulator of CD44. Given these findings and the roles of EHD1 in endocytosis,^16^ we hypothesised that EHD1 might function in the endocytic recycling and lysosomal degradation of the membrane receptor CD44. We found that, after knockdown of EHD1, less CD44 co‐localised with RAB11‐positive recycling vesicles which indicates recycling of endocytosed proteins, but more CD44 co‐localised with lysosome‐associated membrane protein 1 (LAMP‐1, a lysosomal degradation marker) (Figure S9I). Receptor recycling assays showed that the CD44 recycling rate was lower in EHD1^KD^ compared to control cells stimulated with HA for 1 or 2 h (Figure 5G). In addition, a CD44 biotinylation and recycling assay was conducted by ELISA to quantify the total and remaining biotinylated CD44. As shown in Figure 5H and Figure S9J, the inhibitory effect of EHD1 depletion on CD44 recycling was verified in A549 and H1299 cells (Figure 5H and Figure S9J). Conversely, overexpression of WT EHD1 restored CD44 recycling in LUAD cells (Figure S9M).

We considered that the decreased recycling of CD44 to the cell surface in EHD1^KD^ may indicate an increase in posttranscriptional modification and lysosomal degradation of CD44. A cycloheximide (CHX, 20 μg/ml, MCE) chase assay showed that silencing of EHD1 greatly shortened the half‐life of the CD44 protein (Figure 5I and Figure S9K). As expected, treatment with a proteasome inhibitor (MG132, 10 μM, MCE) did not reverse the reduction in the CD44 protein level induced by EHD1 knockdown (Figure 5J), but treatment with a lysosome inhibitor (NH_4_Cl, 10 mM, Aladdin) did (Figure 5K). K63‐linked ubiquitination (UbK63) is critical for lysosomal degradation, whereas K48‐linked ubiquitination (UbK48) facilitates proteasomal degradation.^55^, ^56^ Knockdown of EHD1 increased both the total ubiquitination and UbK63 of CD44 but did not affect UbK48 of CD44 in A549 and H1299 cells (Figure 5L), suggesting that EHD1 protects CD44 from lysosomal degradation but not the proteasomal degradation. On the contrary, EHD1 overexpression inhibited CD44 degradation through suppression of lysosomal activity (Figure S9N).

### 3.6 CD44 silencing reverses the effects of EHD1 on Hippo signalling activity and malignant phenotypes

**FIGURE 6:**
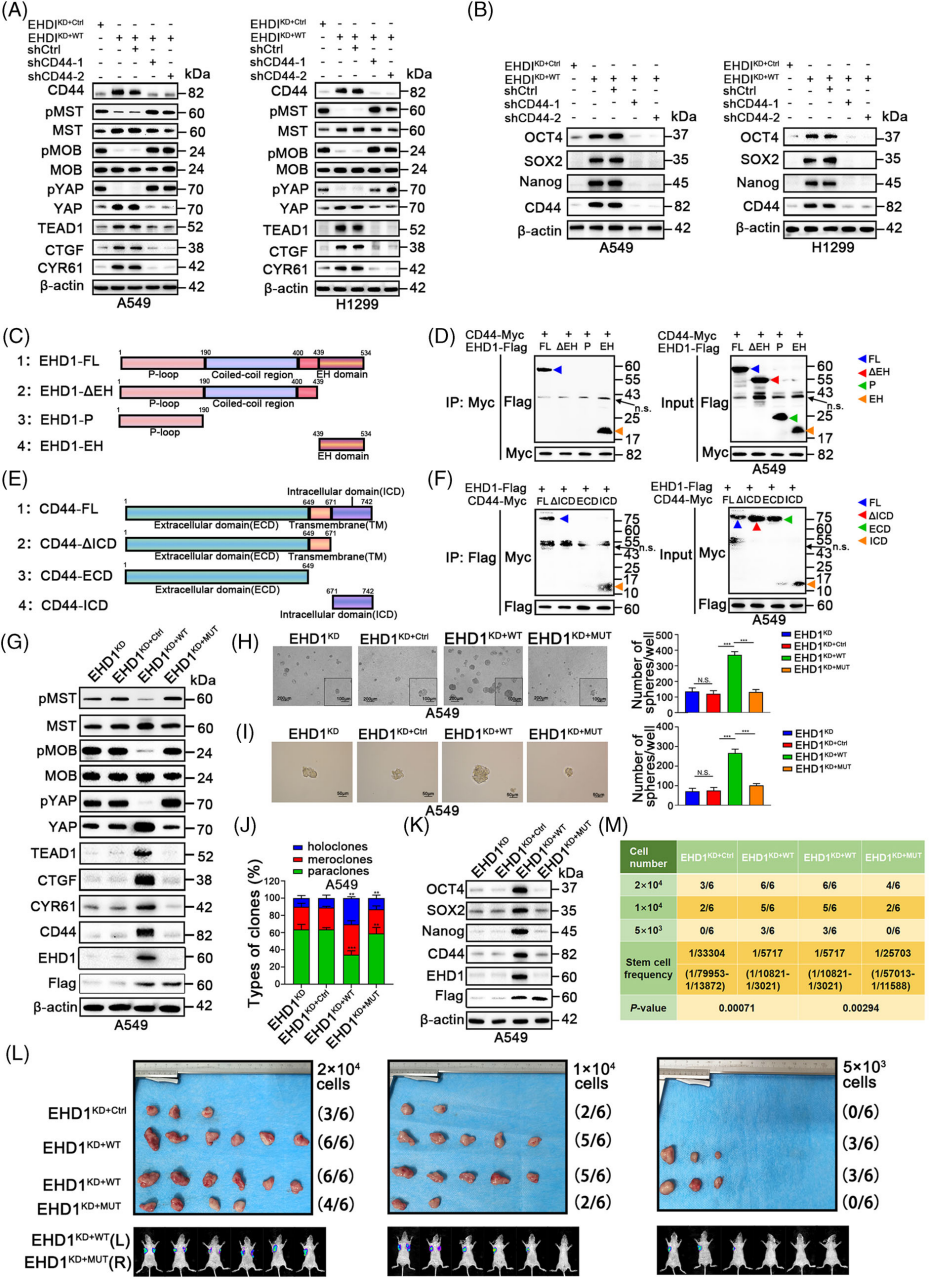
Disruption of the EHD1/CD44 interaction activates the Hippo signalling and attenuates the stemness and metastasis of A549 cells. (A) Expression of core components and downstream targets of the Hippo signalling pathway in LUAD cells. (B) Expression of stemness‐related markers after CD44 knockdown in LUAD cells. (C) Schematic diagram showing the structures of the full‐length EHD1 and deletion constructs used. (D) Flag‐tagged full‐length EHD1 (EHD1‐FL) or EHD1 deletion mutants and Myc‐tagged CD44 were co‐expressed in A549 cells. Extracts were subjected to IP with an anti‐Myc antibody, and bound EHD1 was analysed by Western blotting using an anti‐Flag antibody. n.s.: non‐specific band. (E) Schematic diagram showing the structures of the full‐length CD44 and deletion constructs applied. (F) Myc‐tagged full‐length CD44 (CD44‐FL) or CD44 deletion mutants and EHD1‐Flag were co‐expressed in A549 cells. Extracts were subjected to IP with an anti‐Flag antibody, and bound Myc was analysed by Western blotting using an anti‐Myc antibody. n.s.: non‐specific band. (G) Western blot analysis of the effect of disrupting the EHD1/CD44 interaction on the expression of the core components and downstream molecules of the Hippo signalling pathway. (H and I) The effect of disrupting the EHD1/CD44 interaction on the stemness of A549 cells, as indicated by 3D spheroid cancer models. (J) Holoclone assays showing the effect of disrupting the EHD1/CD44 interaction on stemness. (K) Expression of stemness‐related proteins in A549‐derived cells. (L) Photographs showing the efficiency of tumour generation or tumoursphere formation in nude mice (upper panels). The panels below the images show the bioluminescence signal intensities in nude mice subcutaneously injected with EHD1^KD+Ctrl^ (left) or EHD1^KD+WT^ (right) and nude mice subcutaneously injected with EHD1^KD+WT^ (left) or EHD1^KD+MUT^ (right). (M) The stemness of A549‐derived cells *in vivo* was estimated as the stem cell frequency, with the upper and lower 95% confidence intervals. The data are shown as the mean ± SD values. *p* > .05 was considered N.S., *p < .05, **p < .01 and ***p < .001

Considering the function of CD44 in regulating the Hippo kinase cascade, acquisition of the properties similar to CSCs, EMT progression and metastasis,^53^, ^57^ we speculated that CD44 might be critical for EHD1‐modulated Hippo signalling deactivation, stemness and metastasis. Indeed, CD44 knockdown by shRNAs in EHD1^KD+WT^ effectively restored EHD1‐mediated suppression of Hippo signalling activity (Figure 6A). Consistent with these results, silencing CD44 in LUAD cells significantly attenuated the EHD1‐mediated enhancement of CSCs‐like properties (Figure 6B and Figure S10A–C), invasion, migration and motility (Figure S11A and B) and EMT (Figure S11C). Our data suggest that CD44 mediates the roles of EHD1 in stemness and metastasis via the Hippo pathway in LUAD.

### 3.7 Disruption of the EHD1/CD44 interaction reduces CSCs‐like traits, EMT and metastasis

The EHD1 protein consists mainly of three functional domains: an N‐terminal P‐loop domain, a central coiled‐coil domain and a C‐terminal EH domain. Next, we constructed plasmids expressing Flag‐tagged EHD1‐FL, EHD1‐∆EH, EHD1‐P and EHD1‐EH (Figure 6C) and co‐expressed them with Myc‐tagged CD44. IP assays showed that EHD1‐FL and EHD1‐EH were precipitated by the Myc tag, suggesting that the EH domain is required for the interaction of EHD1 with CD44 (Figure 6D and Figure S12A). Additionally, we found that the cytoplasmic tail of standard CD44 interacted with the EHD1 protein (Figure 6E and F and Figure S12B). Next, EHD1‐∆EH, an *EHD1* mutant without CD44 binding ability, was utilised to establish stable clones (EHD1^KD+MUT^), which were found to lack the regulatory ability of Hippo signalling (Figure 6G and Figure S12C). We demonstrated that disrupting the interaction between EHD1 and CD44 greatly inhibited the EHD1‐mediated enhancement of CSCs‐like characteristics *in vitro* and *in vivo* (Figure 6H–M). In agreement with this observation, disruption of the EHD1/CD44 interaction almost completely abolished the effect of EHD1 on EMT and metastasis of A549 cells (Figure S13A–C). A similar effect on stemness and EMT was also verified in H1299 cells (Figure S12D–G and Figure S13D–F).

### 3.8 EHD1 promotes EMT and metastasis via the CD44/Hippo axis in nude mice

**FIGURE 7:**
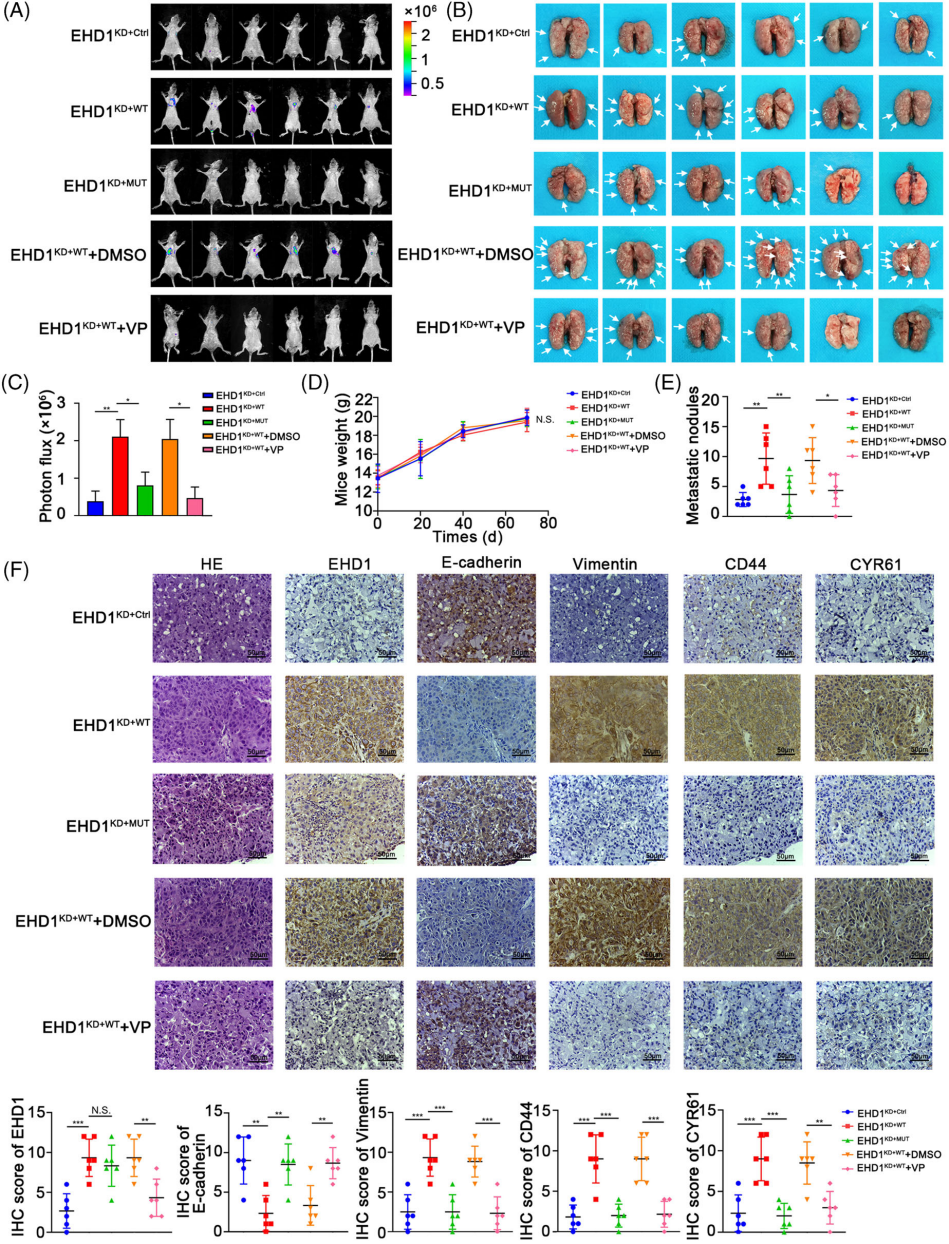
EHD1 promotes metastasis through the CD44/Hippo axis *in vivo*. (A) Bioluminescence images were acquired at the indicated time points. (B) Bar charts showing the results of quantitative and statistical analyses of bioluminescence in lung metastatic foci in all groups. The number of metastatic foci is shown as the mean ± SD; *n* = 6 mice per group. (C) Images of the lungs of nude mice in the groups. The white arrows indicate lung metastatic foci. (D) Statistical analysis of the weights of nude mice in all groups. (E) Statistical analysis of the numbers of metastatic nodules in the indicated lung tissues of nude mice. (F) Representative images of H&E staining and IHC staining for EHD1, E‐cadherin, Vimentin, CD44 and CYR61 in lung tissues from nude mice in all groups (upper panel); The bar charts show the results of quantitative and statistical analyses of the IHC assays (lower panel). The data are shown as the mean ± SD values. *p* > .05 was considered N.S., *p < .05 and **p < .01

Consistent with our prior results indicating that EHD1 knockdown suppressed cancer metastasis to the lung (Figure 2D–G), nude mice intravenously injected with EHD1^KD+WT^ exhibited stronger metastatic bioluminescence signals and more metastatic nodules in the lungs compared with the corresponding controls (Figure 7A–E). To examine whether Hippo signalling activity affects tumour metastasis promoted by EHD1, we treated nude mice with VP or DMSO on day 10 after intravenous injection with EHD1^KD+WT^. VP treatment for 3 weeks diminished the ability of EHD1 to fuel lung metastasis in comparison to DMSO treatment (Figure 7A–E). To test the conclusion that CD44 was essentially for the EHD1‐mediated facilitation of LUAD cell migration and invasion *in vitro*, an *in vivo* experimental approach was performed. Compared with mice subjected to tail vein injection of EHD1^KD+WT^, mice injected with EHD1^KD+MUT^ exhibited decreased metastatic bioluminescence signals and reduced numbers of metastatic nodules in the lungs, indicating that both the EHD1/CD44 interaction and CD44 are important for EHD1‐mediated enhancement of metastasis *in vivo*. The spontaneous lung metastases formed by EHD1^KD+WT^ exhibited a significantly lower levels of E‐cadherin and higher levels of Vimentin, CD44 and CYR61 than those formed by EHD1^KD+Ctrl^ (Figure 7F). Compared to metastases formed by EHD1^KD+WT^, those formed by EHD1^KD+MUT^ displayed an increased expression level of E‐cadherin but decreased expression levels of Vimentin, CD44 and CYR61 (Figure 7F). Inhibition of the Hippo signalling pathway (via VP treatment) exerted a similar influence on the expression of the EMT markers, CD44 and CYR61 with disruption of the EHD1/CD44 interaction (Figure 7F). Our results suggest that EHD1 might efficiently potentiate EMT and metastasis by regulating the CD44/Hippo signalling pathway *in vivo*.

### 3.9 A positive feedback loop exists between EHD1 and the previously reported CD44/Hippo/SP1 axis

Intriguingly, during this study, we observed that treatment with VP, a disruptor of the YAP‐TEADs interaction, inhibited EHD1 expression (Figure 4A and Figure S7A). Hence, we hypothesised the possible existence of a positive feedback loop, such as Hippo signalling inactivation that can also regulate EHD1 expression. We first investigated whether EHD1 expression is a direct transcriptional output of Hippo signalling. JASPAR analysis (http://jaspar.genereg.net) indicated that TEAD transcription factor (TF) family members (TEAD1‐4) were not enriched in the EHD1 promoter region. Previous studies showed that, in addition to the TFs acting individually, numerous TFs are assembled into densely connected transcriptional networks with a robust capacity to regulate genes involved in cancer development.^58^, ^59^ Thus, we assumed that there might be TFs that are both upstream TFs of EHD1 and downstream target genes of TEADs.

**FIGURE 8:**
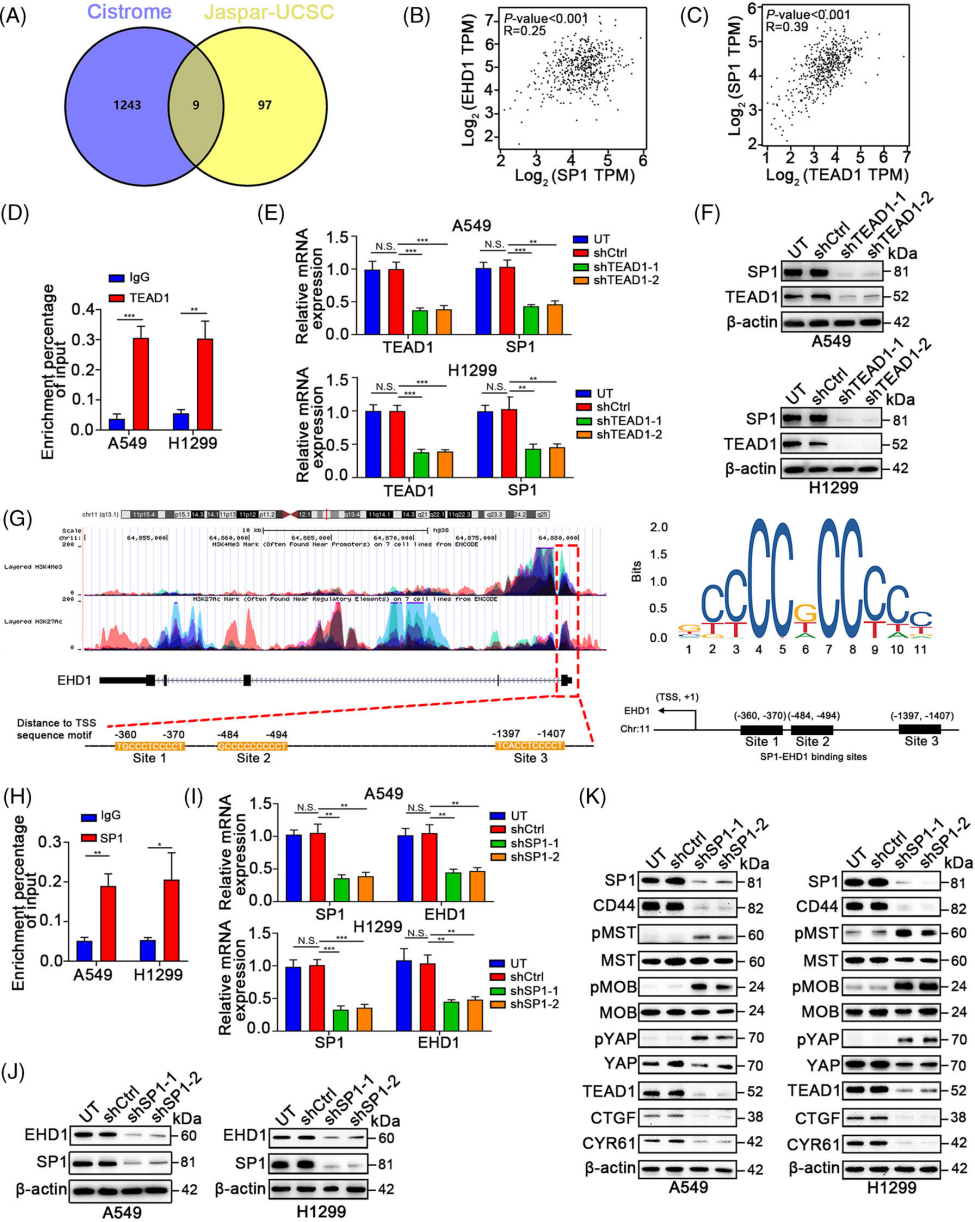
SP1 promotes the transcription of EHD1. (A) Venn diagram showing the overlapping targets transcriptionally regulated by TEAD1 and the TFs of EHD1 identified in JASPAR (http://jaspar.genereg.net), the UCSC Genome Browser (https://genome.ucsc.edu/) and Cistrome Data Browser (http://cistrome.org/db/#/). (B) The correlation between SP1 and EHD1 mRNA expression was analysed on the GEPIA website (http://gepia.cancer‐pku.cn/). (C) The correlation between TEAD1 and SP1 mRNA expression was analysed on the GEPIA website. (D) ChIP‐PCR validated the binding of TEAD1 in the SP1 promoter. The regulation of TEAD1 knockdown on SP1 expression which was assessed by qRT‐PCR (E) and Western blotting (F). (G) The UCSC Genome Browser website showed that aberrant histone modifications were highly enriched at the promoter of EHD1, which accounted for its efficient activation. The SP1 binding motif and three potential SP1‐specific NBSs were predicted to exist in the promoter region of the *EHD1* gene by the JASPAR database. (H) qRT‐PCR analysis of the ChIP products validated the binding ability of SP1 to the EHD1 promoter. (I) The effect of SP1 knockdown on the transcription of EHD1 was assessed by qRT‐PCR. (J) The effect of SP1 knockdown on the EHD1 protein level was estimated by Western blotting. (K) The effects of SP1 knockdown on CD44 expression and Hippo signalling pathway activity were assessed by Western blotting. The data are shown as the mean ± SD values. *p* > .05 was considered N.S., *p < .05, **p < .01 and ***p < .001

To identify the TFs, online bioinformatics databases, including JASPAR, UCSC Genome Browser (https://genome.ucsc.edu/) and Cistrome Data Browser (http://cistrome.org/db/#/), were applied. As shown in Figure 8A and Figure S14A, nine TFs were predicted. Among them, only SP1 has been reported to be transcriptionally regulated by TEAD1 via direct binding of TEAD1 to the promoter of SP1.^60^ Herein, SP1 was preferentially selected for verification of its status as the TF of EHD1. Moreover, a positive significant correlation between the mRNA expression levels of SP1 and EHD1 was observed in tissue samples from The Cancer Genome Atlas (TCGA) LUAD cohort (Figure 8B). In this cohort, the mRNA expression of TEAD1 was positively correlated with that of SP1 (Figure 8C). Consistent to the previous study in colorectal cancer,^60^ TEAD1 could promote SP1 transcriptional activation and expression in LUAD cells (Figure 8D–F).

Furthermore, analysis of the online UCSC Genome Browser showed that abnormal histone modifications, namely, histone 3 Lys4 trimethylation (H3K4me3) and histone 3 Lys27 acetylation (H3K27Ac), were highly enriched at the promoter of EHD1, which accounted for the efficient activation of EHD1; moreover, we found three potential SP1‐specific nucleotide‐binding sites (NBSs) (Figure 8G). Chromatin immunoprecipitation (ChIP) assay demonstrated that SP1 directly bound to the EHD1 promoter (Figure 8H). qRT‐PCR and Western blot analyses revealed that SP1 knockdown suppressed EHD1 expression at both the transcript (Figure 8I) and protein (Figure 8J) levels.

In line with the results of previous studies, we observed that SP1 silencing reduced stemness features (Figure S14B–E) and inhibited EMT and metastasis (Figure S15) in A549 and 1299 cells. As expected, we found that SP1 can function as a regulator of Hippo signalling activity and CD44 expression (Figure 8K), suggesting that SP1 might play a pivotal role in promoting stemness properties and LUAD metastasis via the CD44/Hippo axis. Collectively, these results indicated the existence of a positive feedback loop between EHD1 and the previously established CD44/Hippo/SP1 axis.

### 3.10 EHD1 is positively correlated with CD44, CTGF and SP1 in clinical LUAD specimens

**FIGURE 9:**
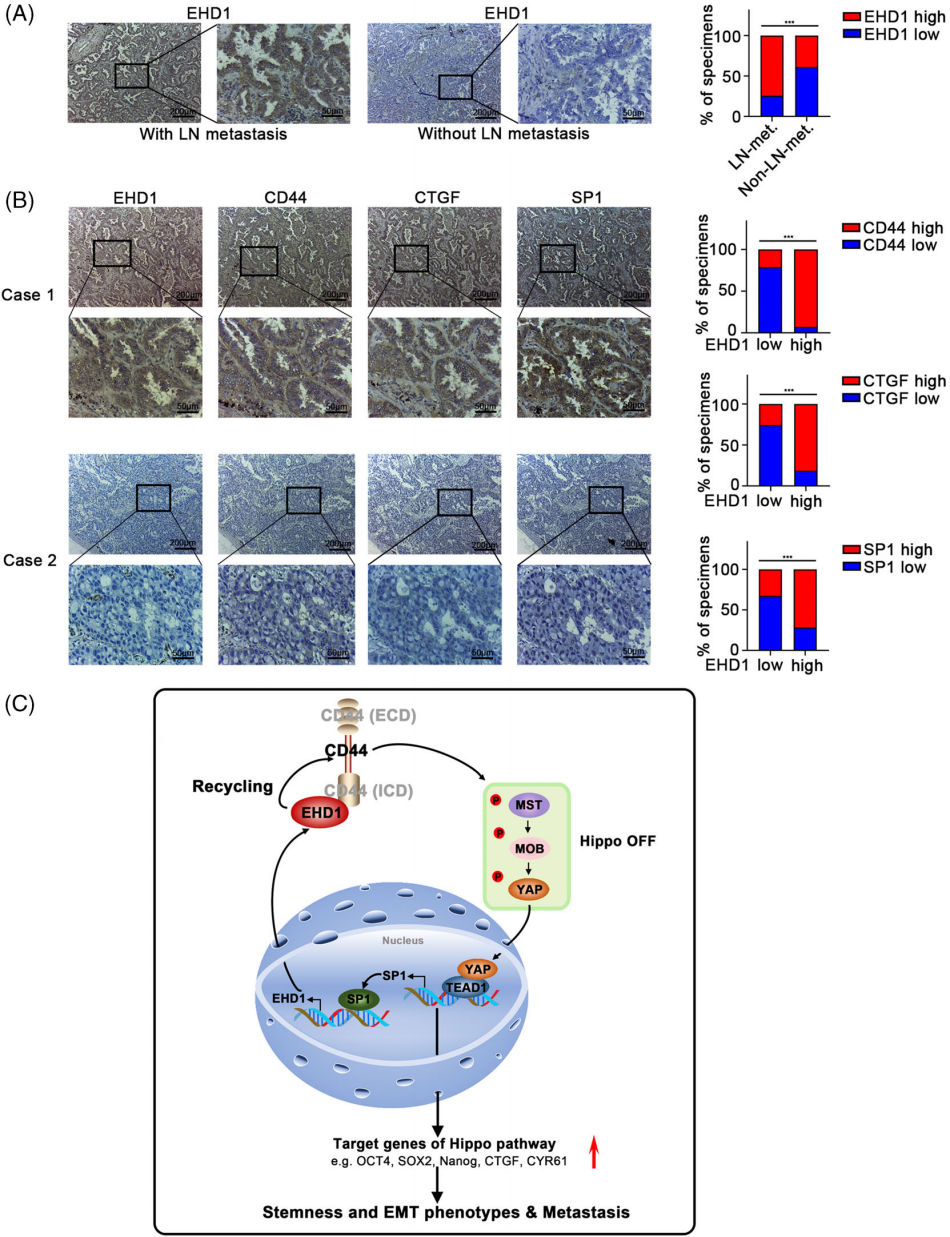
Associations among EHD1, CD44, CTGF and SP1 expression in LUAD specimens. (A) Representative immunostaining profiles of EHD1 expression in LUAD tissues of the patients with lymph node metastasis (*n* = 58) and in LUAD tissues of the patients without lymph node metastasis (*n* = 55, left panel). The bar graphs show the statistical analysis of EHD1 expression in these two groups (right panel). The data are shown as the mean ± SD values. ***p < .001. (B) Representative images of immunohistochemical staining for EHD1, CD44, CTGF and SP1 in serial sections of LUAD samples from patients. Case 1 is representative of a LUAD patient with high EHD1 expression, whereas Case 2 is representative of a LUAD patient with low EHD1 expression. The bar graphs show that EHD1 expression determined by IHC was associated with CD44, CTGF or SP1 expression in 113 clinical LUAD specimens. The data are shown as the mean ± SD values. ***p < .001. (C) Schematic representation of the EHD1/CD44/Hippo/SP1 positive feedback loop in metastatic LUAD. EHD1 can interact with the CD44‐ICD, promoting CD44 recycling and upregulating CD44 protein expression. Subsequently, CD44 upregulation inhibits Hippo signalling activity, leading to nuclear entry of dephosphorylated YAP as well as a subsequent increase in the expression of downstream gene targets (i.e. OCT4, SOX2, Nanog, CTGF and CYR61, which are related to stemness and/or EMT). Thus, EHD1 antagonises the Hippo pathway to enhance the CSCs‐like properties, EMT and metastasis of LUAD cells. Moreover, SP1, as a downstream target of Hippo signalling, in turn transcriptionally activates EHD1 expression, finally forming a positive feedback loop that drives LUAD metastasis

To detect the relationship between EHD1 expression and lymphatic metastasis, we collected LUAD tissue samples from 113 patients who treated with removal sections in Harbin Medical University Cancer Hospital. According to the results of IHC assays, the EHD1 expression level was higher in LUAD tissues of patients with lymph node metastasis than in those without (Figure 9A). Our IHC results suggested that EHD1 expression might be positively associated with tumour metastasis. Consistent with our above observations, EHD1 expression was positively linked with CD44, CTGF and SP1 expression in LUAD tissue specimens (Figure 9B).

## 4 DISCUSSION

Most LUAD patients do not succumb to their primary cancer but instead succumb to cancer metastasis.^61^ The clinically relevant mechanisms have not been fully elucidated. Herein, we identified a significantly prognostic EMG, EHD1. Moreover, our *in vitro* and *in vivo* data revealed a novel regulatory mechanism controlling LUAD metastasis in which EHD1, CD44, Hippo signalling and SP1 form a positive feedback loop. In detail, EHD1 can interact with CD44‐ICD, promoting CD44 recycling and upregulating CD44 protein expression. Subsequently, the upregulated CD44 inhibits Hippo signalling activity, leading to nuclear entry of dephosphorylated YAP as well as a subsequent increase in the expression of downstream target genes (i.e. OCT4, SOX2, Nanog, CTGF and CYR61, which are related to stemness and/or EMT).^51^, ^62^ Thus, EHD1 antagonises the Hippo pathway to enhance the CSCs‐like properties, EMT and metastasis of LUAD cells. Moreover, SP1, as a downstream target of Hippo signalling, in turn transcriptionally activates EHD1 expression, finally forming a positive feedback loop that drives LUAD metastasis (Figure 9C).

In agreement with our findings, recent studies have suggested that high EHD1 expression predicts a poor prognosis in lung cancer,^32^, ^33^ osteosarcoma^23^ and breast cancer,^25^ suggesting that EHD1 could serve as a potential therapeutic target. Similar to our results, previous studies have supported the roles of EHD1 in regulating EMT and stemness properties. Huang et al. revealed that EHD1 modulates microtubule stability via interacting with Class III β‐tubulin, which activates the PTEN/PI3K/AKT signalling pathway and then induces EMT and the resistance of EGFR‐tyrosine kinase inhibitor (EGFR‐TKI).^63^ EHD1 is reported to be upregulated by the nuclear factor kappa‐B/miR‐590 axis and to enhance CSCs‐like properties and EGFR‐TKI resistance.^64^

The attractive concept of CSCs, which holds that this population of cancer cells endowed with self‐renewal and tumour‐seeding potential could drive cancer initiation and progression, has been widely recognised.^62^, ^65^ Importantly, this concept provides an explanation for cancer heterogeneity that confers resistance to chemotherapeutic drugs, radiotherapy, targeted therapy and immunotherapy, accounting for the poor survival of LUAD patients.^66^ Our previous studies have suggested that EHD1 plays roles in chemoresistance and EGFR‐TKI resistance.^31^, ^35^, ^36^ The speculation that EHD1 regulates resistance to other types of therapies through CSCs‐like properties deserves further investigation.

In this study, EHD1 was identified for the first time as a robust regulator of the Hippo kinase cascade. When Hippo signalling is activated, the complex comprising phosphorylated MST1/2 and SAV1 further phosphorylates and activates the LATS1/2‐MOB1 complex, leading to the phosphorylation, cytoplasmic retention and eventual ubiquitination and degradation of YAP/TAZ.^67^ Accumulating evidence has confirmed that Hippo signalling controls the transcription of numerous target genes to play essential roles in cancer progression.^51^ Therefore, Hippo‐targeted therapy is considered an attractive approach.^68^ In a Phase 0 clinical trial, Visudyne, an FDA‐approved form of VP, was effectively absorbed into cancer cells of human patients.^69^ In preclinical glioblastoma models, there was a survival benefit of VP at nontoxic levels.^70^ Here, VP administration significantly impaired EHD1‐mediated malignant behaviour *in vitro* and in mouse models. Our current results might aid in the development of better techniques for selecting LUAD patients most likely to benefit from Hippo‐targeted therapy.

Our study is the first to reveal that EHD1 promotes the endocytic recycling of CD44, thus preventing its lysosomal degradation. During the endocytic trafficking process, internalised cargos, including integral receptors and their associated ligands, face two destinies: entry into either the endocytic recycling compartment (ERC) for endocytic recycling or entry into the lysosome for degradation.^17^, ^71^ In mammalian cells, EHD1 acts as a director of the endocytic trafficking and recycling of internalised cargos.^16^ Consistent with our hypothesis that EHD1 is implicated in the endocytic trafficking of CD44, we revealed that EHD1 increases CD44 cell surface expression and recycling. Moreover, EHD1 was demonstrated to play critical roles in several steps of the endocytic recycling of plasma membrane components from early endosomes (EEs, also called sorting endosomes) to the ERC and from the ERC to the plasma membrane, retrograde transport of cargos from the EE to the Golgi and endosome‐derived vesicular fission processes.^18^, ^19^, ^20^, ^21^, ^72^, ^73^, ^74^, ^75^, ^76^ For instance, EHD1 binding to the RAB11 effector RAB11‐FIP2 modulates the trafficking of RAB11‐positive vesicles from the ERC to the plasma membrane.^74^ Dynein motors drive EE‐to‐ERC transit, and EHD1 is related to them.^75^ Specifically, elucidating which step(s) in CD44 endocytic recycling are regulated by EHD1 will be an interesting future pursuit.

Currently, TFs account for approximately 20% of all identified oncogenes.^77^ Consistent with previous studies,^78^, ^79^, ^80^ our study showed that knockdown of the TF SP1 reduces CSCs‐like traits, EMT and metastasis of LUAD cells. SP1 can transcriptionally activate and repress numerous crucial oncogenes and tumour suppressors.^80^ Moreover, we discovered that SP1 promotes EHD1 transcriptional activation, accompanied by regulation of CD44 and the Hippo signalling pathway. Three putative SP1‐specific NBSs were predicted to be located in the EHD1 promoter region; however, which of the NBSs is required for transcriptional activation of EHD1 needs to be identified.

In summary, our data reveal that EHD1 orchestrates Hippo signalling‐mediated CSCs‐like traits, EMT and metastasis by regulating CD44 recycling and stability. SP1, a reported target of Hippo signalling, transcriptionally activates EHD1 expression, forming an EHD1/CD44/Hippo/SP1 positive feedback loop. Eventually, this loop leads to persistent Hippo signalling deactivation and LUAD metastasis. These findings might provide a more comprehensive understanding of the regulatory mechanisms driving the coupling modules of stemness and EMT. Additionally, our work indicates that targeting EHD1 and blocking this feedback loop might be promising therapeutic options for metastatic LUAD.

## COMPETING INTERESTS

The authors declare that they have no competing interests.

## Supporting information

Supporting information

Supporting information

Supporting information

Supporting information

Supporting information

Supporting information

Supporting information

Supporting information

Supporting information

Supporting information

Supporting information

Supporting information

Supporting information

Supporting information

Supporting information

Supporting information

Supporting information

Supporting information

Supporting information

Supporting information
