# Supplementary material for: A novel EHD1/CD44/Hippo/SP1 positive feedback loop potentiates stemness and metastasis in lung adenocarcinoma
Source: Clin Transl Med. 2022 Apr 29;12(4):e836. doi: 10.1002/ctm2.836 (PMC9786223; doi:10.1002/ctm2.836)
Supplement: Supplementary file 1 — Supporting information [file CTM2-12-e836-s011.docx]

**A** **novel EHD1/CD44/Hippo/SP1 positive feedback loop potentiates stemness and metastasis in lung adenocarcinoma**

Yuechao Liu^1†^, Yang Song^2†^, Mengru Cao^1^, WeiNa Fan^1^, Yaowen Cui^1^, Yimeng Cui^1^, Yuning Zhan^1^, Ruixue Gu^1^, Fanglin Tian^1^, Shuai Zhang^1^, Li Cai^1*^, Ying Xing^1*^

^†^ Yuechao Liu and Yang Song contributed equally to this work.

1 The Fourth Department of Medical Oncology, Harbin Medical University Cancer Hospital, 150 Haping Road, Harbin, 150081, China;

2 The First Department of Orthopedic Surgery, The Second Afﬁliated Hospital of Harbin Medical University, Harbin, 150086, China

**Supplementary materials and methods**

**Bioinformatic analysis and datasets**

Gene expression data profiled in the GEO database (GSE175601) was downloaded to conduct differential analysis (*P* < 0.01 and absolute fold change > 2). A total of 181 endocytic genes were obtained from Gene Set Enrichment Analysis (GSEA, <http://www.gseamsigdb.org/gsea/msigdb/geneset_page.jsp?geneSetName=KEGG_ENDOCYTOSIS&keywords=Endocytosis>). To investigate the prognostic significance of EMGs, Kaplan-Meier analyses were conducted by the survminer R package under the R-platform.

**qRT-PCR**

In brief, total RNA was obtained using a RNeasy kit (Omega, USA) and was reverse transcribed into cDNA using a Transcriptor cDNA Synthesis Kit (Roche, Germany). qRT-PCR was performed in a 7500HT Fast Real-Time PCR System (Applied Biosystems). The cycle threshold (Ct) value of each sample was determined, and qPCR data were analyzed by a two-step method. GAPDH was used as the internal control. The primers used for qRT-PCR are listed in Table S3.

**Western blot analysis**

Protein obtained from LUAD cells was homogenized and separated on SDS-PAGE gels (Beyotime, China). The electrophoresis products were transferred onto PVDF membranes. Membranes were blocked with 5% skim milk and incubated with primary antibodies at 4 °C overnight. After incubation with HRP-conjugated secondary antibodies at 25 ℃ for 1 h, membranes were visualized in a FluorChem E Ultrasensitive Automatic Imaging Analysis System (Protein Simple, USA) according to the instructions. The antibodies used for Western blot analysis s are listed in Table S4.

***In* *vitro* ELDA**

LUAD cells were seeded at a density of 50, 100 or 200 cells per well in 96-well plates, and then the stem cell frequency was then analyzed after 7 days. The stem cell frequency was calculated with ELDA software (http://bioinf.wehi.edu.au/software/elda/).

**Wound healing assay**

Wound healing assay was used to examine the migratory ability and motility of LUAD cells. Approximately 10^6^ LUAD cells were seeded into each well of a 6-well plate. The next day, two perpendicular wounds were drawn in a straight line along a ruler with a 200 μl pipette tip. The pipette tip remained vertical and was not tilted. After scratching, the cells were washed with PBS to remove the unattached cells. Next, 3 ml of serum-free RPMI 1640 medium was added to each well, and images were acquired at the 0 h time point. Then, the plates were incubated at 37 °C in 5% CO_2_ for 24 h. Subsequently, images were acquired at 24 h. Next, the cells in the wells were fixed with 800 μl of 4% paraformaldehyde solution for 1 h and were then stained with 600 μl of 0.1% crystal violet for 2 h. Finally, the wells were washed with PBS and imaged again. Normalized invasion cell or migration cell number = actual invasion cell or migration cell number/cell growth rate.

**Transwell assay**

Transwell assays were used to examine the migratory and invasive abilities of LUAD cells. Thirty microliters of diluted Matrigel (serum-free RPMI 1640 cell culture medium: Matrigel=7:1) was added to the upper compartment of a Transwell chamber and incubated in a 37 °C incubator for 2 h. Chambers with a Matrigel-coated membrane were considered the invasion environment for assessment of the cell invasion ability, and chambers without a Matrigel coating on the membrane were used to evaluate the cell migration ability. LUAD cells were harvested by trypsin digestion, and the trypsin was then neutralized with serum-free RPMI 1640 medium. Next, 200 μl of the single-cell suspension was added to the upper compartment, and 600 μl of cell culture medium containing 10% FBS was added to the lower compartment. Then, the Transwell plates were incubated for 24 h in a 37 °C incubator for evaluation of cell migration. In addition, to determine the invasive capability of cells, after incubation for 48 h, the passage of cells through the lower surface of the porous membrane was evaluated. The noninvaded cells on the upper surface of the membrane were gently removed by wiping. Then, the cells on the membranes were fixed with 4% paraformaldehyde solution and stained with 0.1% crystal violet. Finally, the membranes were washed with PBS, and images were acquired with a microscope (Leica, Germany) for cell counting. Normalized invasion cell or migration cell number = actual invasion cell or migration cell number/cell growth rate.

**Immunofluorescence assay**

Cells were digested after centrifugation and counted. A total of 10^4^ cells were added and incubated in confocal dishes in an incubator at a constant temperature of 37 °C overnight. The dishes were removed the next day and washed 3 times in PBS. Next, 4% paraformaldehyde solution was used to fix the cells for 15 min. Then, the cells were permeabilized with 0.5% Triton X-100 solution in PBS for 20 min. Subsequently, the cells were blocked with goat serum for 30 min, and the primary antibody was added directly. The dishes were placed in a refrigerator overnight at 4 ℃. The next day, the confocal dishes were transferred to a 25 ℃ environment. The cells were then rinsed three times with PBST and incubated with fluorescent secondary antibodies for 1 h at 37 °C. Subsequently, the cells were stained with DAPI reagent (Boster, China) for 5 min after 3 washes in PBST. Finally, the cells were visualized with a confocal microscope (Nikon). The antibodies used in the immunofluorescence assay were as follows: anti-YAP (Abcam, ab52771), anti-EHD1 (Abcam, ab109747), anti-CD44 (Abcam, ab6124), anti-Rab11 (Proteintech, 15903-1-AP), and anti-LAMP-1 (Proteintech, 55273-1-AP).

**Coimmunoprecipitation (Co-IP)**

Endogenous and exogenous Co-IP experiments were performed with a Pierce™ Crosslink Magnetic IP/Co-IP Kit (Thermo Fisher Scientific, #88805). Pierce protein A/G magnetic beads were mixed with anti-Myc, anti-Flag, anti-EHD1 and anti-CD44 antibodies or IgG at 25 °C in a thermostatic rotator for 2 h, and the protein extracts from LUAD cells were lysed in IP Lysis/Wash Buffer at 4 °C in a thermostatic rotator overnight. Then, the proteins bound to the magnetic beads were washed off with eluent buffer. Next, the proteins were boiled for 5 min after adding SDS buffer prior to the further steps of Western blot analyses.

**Immunohistochemistry (IHC)**

First, the tissues on the slides were confirmed to be tumor tissues by H&E staining. Serial paraffin sections (4 μm thick) of mouse lung tissues were stained with antibodies specific for EHD1 (Abcam, ab109311), CD44 (Proteintech, 60224-1-Ig), E-cadherin (Proteintech, 20874-1-Ig), Vimentin (Proteintech, 60330-1-Ig) and CYR61 (Proteintech, 67656-1-Ig). The percentage of positive cells (% of PPs) and the staining intensity (SI value) were obtained and multiplied to calculate the immunoreactive score (IRS value).^1^

**Chromatin immunoprecipitation (ChIP) analysis**

An Enzymatic Chromatin IP Kit (Magnetic Beads, CST, #9003) was used to perform the ChIP assay. In brief, cells were crosslinked with 1% formaldehyde at 25 °C for 10 min, and glycine was used to inactivate the excess formaldehyde. Then, anti-RNA polymerase, nonspecific IgG, anti-TEAD1 and anti-SP1 antibodies were added to the chromatin extracts. Next, the chromatin-containing DNA fragments were subjected to IP. Subsequently, the DNA was decrosslinked, and the bound TEAD1-SP1 and SP1-EHD1 complexes were analyzed by qRT-PCR. The primer pairs used for PCR amplification are listed in Table S3.

**Statistical analysis**

The experimental data were analyzed by *t* tests, ANOVA and other methods to determine differences among the experimental groups. A *P* value < 0.05 was considered to indicate statistical significance. Moreover, all data were collated, and graphs were produced with GraphPad 8.0 software. All experiments were repeated at least three times. The data are shown as the mean ± standard deviation (SD) values.

**Reference**

1. Wang KJ, Wang C, Dai LH, Yang J, Huang H, Ma XJ, et al. Targeting an Autocrine Regulatory Loop in Cancer Stem-like Cells Impairs the Progression and Chemotherapy Resistance of Bladder Cancer. Clin Cancer Res. 2019;25(3):1070-86.

**Supplementary figure legends**

**FIGURE S1** EHD1 knockdown inhibits the stemness of H1299 cells. (A-B) EHD1 knockdown in H1299 cell lines was examined by qRT-PCR and Western blot analysis. (C-E) The effect of EHD1 depletion on the CSCs-like traits of H1299 cells was determined *in vitro*. The (C) 3D spheroid assays using semisolid medium, (D) 3D spheroid assays using serum-free medium and (E) holoclone assays showed the CSCs-like traits of H1299 cells. (F) The expression of stemness-related markers in H1299 cells. (G) In the *in vitro* limiting dilution assays, the numbers of wells in 96-well plates that contained tumor spheres were determined (upper panel). The stemness of H1299 cells with or without EHD1 knockdown was estimated as the stem cell frequency (bottom panel). The data are shown as the mean ± SD values. *P* > 0.05 was considered not significant (N.S.), **P* < 0.05, ***P* < 0.01 and ****P* < 0.001.

**FIGURE S2** EHD1 overexpression promotes the stemness of A549 cells. (A-B) Overexpression of EHD1 was examined by qRT-PCR and Western blot analysis. (C-E) The effect of EHD1 restoration on the stemness of LUAD cells was determined *in vitro*. The (C) 3D spheroid assays with semisolid medium, (D) spheroid assays with stem cell medium and (E) holoclone assays showed the CSCs-like traits of A549 cells. (F) Expression of stemness-related proteins in A549 cells. (G) Flow cytometric analysis of CD133- and CD44-positive cells. (H) In the *in vitro* limiting dilution assays, the numbers of wells in 96-well plates that contained tumor spheres were determined (upper panel). The stemness of A549 cells with or without EHD1 overexpression was estimated as the stem cell frequency (lower panel). The data are shown as the mean ± SD values. *P* > 0.05 was considered N.S., **P* < 0.05, ***P* < 0.01 and ****P* < 0.001.

**FIGURE S3** EHD1 restoration enhances the stemness of H1299 cells. (A-B) The 3D spheroid cancer models showed the effect of EHD1 overexpression on stemness. The bar graphs show the quantification of the number of spheres per well formed by H1299-derived cells. (C) The stemness of H1299 cells with or without EHD1 overexpression was determined by holoclone assays. (D) Expression of stemness-related proteins in H1299 cells. (E) In the *in vitro* limiting dilution assays, the number of tumor spheres in each well of 96-well plates was determined (top panel). The stemness of H1299 cells with or without EHD1 overexpression was estimated as the stem cell frequency (bottom panel). The data are shown as the mean ± SD values. **P* < 0.05, ***P* < 0.01 and ****P* < 0.001.

**FIGURE S4** Knockdown of EHD1 suppresses the metastasis of H1299 cells. (A) Representative images of wound healing assays showing the effect of EHD1 knockdown on the migration ability and motility of H1299 cells (left panel). Quantitative and statistical analyses based on the wound area depicted cell migration and motility (right panel). (B) Transwell assays determined the effect of EHD1 knockdown on the migration and invasion of H1299 cells. (C-D) The morphology change was induced by EHD1 knockdown in A549 and H1299 cells. (E) The expression of EMT-related markers in H1299 cells after EHD1 knockdown. The data are shown as the mean ± SD values. *P* > 0.05 was considered N.S., ***P* < 0.01 and ****P* < 0.001.

**FIGURE S5** Overexpression of EHD1 accelerates LUAD cells metastasis. (A) Representative images of wound healing assays showed the effect of EHD1 overexpression on the migration ability and motility of A549 cells (left panel). Quantitative and statistical analyses based on the wound area showing cell migration and motility (right panel). (B) Transwell assays detected the effect of EHD1 overexpression on the migration and invasion of A549 cells. (C) The morphology change was caused by EHD1 overexpression in A549 cells. (D) Expression of EMT-related markers in A549 cells after EHD1 restoration. (E) Wound healing assays determined the effect of EHD1 overexpression on the migration ability and motility of H1299 cells. (F) Transwell assays detected the effect of EHD1 overexpression on the migration and invasion of H1299 cells. (G) The morphology change was initiated by EHD1 overexpression in H1299 cells. (H) Expression of EMT-related markers in H1299 cells after EHD1 restoration. The data are shown as the mean ± SD values. ***P* < 0.01 and ****P* < 0.001.

**FIGURE S6** EHD1 inactivates the Hippo signaling pathway in H1299 cells. (A) Relative IF images showing the localization of YAP in H1299-derived cells. (left panel). Green indicates YAP IF staining; blue indicates DAPI staining. Statistical analysis of YAP localization in H1299 cells is shown in the bar graphs (right panel). (B) The expression levels of YAP in the cytoplasm and nucleus were determined by subcellular fractionation assays in H1299-derived cells. β-actin was used as the control for cytoplasmic expression, while Lamin B was used as the control for nuclear expression. (C) Luciferase reporter assays showing YAP transcriptional activity in NC and EHD1^KD^ transfected with the empty vector or YAP plasmid. (D) Expression of core components and downstream targets of the Hippo signaling pathway in H1299 cells. (E) Representative IF images validating the localization of YAP in H1299 cells with or without EHD1 overexpression (left panel). Bar graphs showing the statistical analysis of YAP localization in H1299 cells (right panel). (F) The expression levels of YAP in the cytoplasm and nucleus of H1299 cells with or without EHD1 restoration were determined by subcellular fractionation assays. β‐actin was used as the control for cytoplasmic expression, while Lamin B was used as the control for nuclear expression. (G) Luciferase reporter assays determined YAP transcriptional activity in H1299 cells with or without EHD1 restoration that were transfected with the empty vector or YAP plasmid. (H) The expression of core components and downstream targets of the Hippo signaling pathway was detected by Western blotting. The data are shown as the mean ± SD values. **P* < 0.05, ***P* < 0.01 and ****P* < 0.001.

**FIGURE S7** Hippo signaling is essential for EHD1-mediated enhancement of metastasis and stemness in H1299 cells. (A) Western blot analysis validated the effect of treatment with VP (1 μM) or DMSO for 24 h on the Hippo signaling pathway in the EHD1^KD+WT^ group. The expression of core components and downstream targets of the Hippo signaling pathway was detected. (B-C) The 3D spheroid cancer models showed the effect of VP treatment on stemness in the EHD1^KD+WT^ group. The bar graphs show the quantification of the number of spheres per well formed by H1299-derived cells. (D) The stemness of H1299 cells treated with DMSO or VP was evaluated by holoclone assays. (E) Expression of stemness-related proteins in H1299-derived cells treated with DMSO or VP. (F) Wound healing assays showed the effect of VP treatment on the migration ability and motility of H1299-derived cells. (G) Transwell assays detected the effect of DMSO or VP treatment on the migration and invasion of H1299 cells. (H) Expression of EMT-related markers in H1299-derived cells treated with DMSO or VP. The data are shown as the mean ± SD values. *P* > 0.05 was considered N.S., **P* < 0.05, ***P* < 0.01 and ****P* < 0.001.

**FIGURE S8** Hippo signaling is essential for EHD1-mediated enhancement of A549 cell metastasis. (A) Wound healing assays showed the effect of VP treatment on the migration ability and motility of EHD1^KD+WT^. (B) Transwell assays validated the effect of DMSO or VP treatment on the migration and invasion of A549 cells. (C) Expression of EMT-related proteins in A549 cells treated with DMSO or VP. The data are shown as the mean ± SD values. *P* > 0.05 was considered N.S. and ***P* < 0.01.

**FIGURE S9** EHD1 interacts with CD44 and enhances its stability. (A) Venn diagram indicating that 242 proteins were identified in the IgG control group and 266 proteins were identified in the EHD1 experimental group, among which 88, including EHD1, CD44 and other proteins, specifically bound to EHD1. (B) Identification of EHD1 peptides by MS (red indicating identified amino acid). (C) Expression of phosphorylated and total Merlin after EHD1 knockdown in LUAD cells. (D-F) Endogenous (D-E) and exogenous (F) IP experiments validated the interaction of EHD1 and CD44. (G) The mRNA level of CD44 in LUAD cell lines with EHD1 knockdown was examined by qRT-PCR. (H) Western blot analysis was performed to examine the effect of EHD1 knockdown on the regulation of CD44 protein expression. (I) Colocalization analysis of CD44 with RAB11 and LAMP-1 before or after 1 h HA treatment in control and EHD1-knockdown LUAD cells. (J) The biotinylation and recycling assay of CD44 by ELISA showed CD44 recycling in NC and EHD1^KD^ H1299 cells. (K) A CHX chase assay was performed to analyze the half-life of the CD44 protein in NC and EHD1^KD^ H1299 cells. Cells were incubated in the presence of CHX (20 μg/ml) for 0, 1, 2, 3, or 4 h. (L) The MFI of cell relative surface CD44 was detected by FACS. The relative surface level is related to the amount of the receptor that undergoes ligand fixation and stimulation, internalization and recycling to the cell surface. Representative flow cytometry data and statistical analysis of cell surface CD44 in A549 (left) and H1299 (right) cells. (M) Biotinylation and recycling assays of CD44 by ELISA showed CD44 recycling in EHD1^KD+Ctrl^ and EHD1^KD+WT^ in LUAD cells. (N) CD44 ubiquitination assays in LUAD cells transfected with the Total-Ub, K63-Ub or K48-Ub plasmid after treatment with NH_4_Cl for 8 h. The data are shown as the mean ± SD values. *P* > 0.05 was considered N.S., **P* < 0.05, ***P* < 0.01 and ****P* < 0.001.

**FIGURE S10** CD44 depletion inhibits the stemness of LUAD cells. (A-C) The effect of CD44 silencing on the stemness of LUAD cells was determined *in vitro*. The (A) 3D spheroid assays using semisolid medium, (B) 3D spheroid assays using serum-free medium and (C) holoclone assays showed the CSCs-like traits of LUAD cells. The data are shown as the mean ± SD values. *P* > 0.05 was considered N.S., **P* < 0.05, ***P* < 0.01 and ****P* < 0.001.

**FIGURE S11.** CD44 knockdown suppresses LUAD cells metastasis. (A) Wound healing assays showed the effect of CD44 knockdown on the migration ability and motility of LUAD cells. (B) Transwell assays showed the effect of CD44 knockdown on the migration and invasion of LUAD cells. (C) Expression of EMT-related markers in LUAD cells after CD44 knockdown. The data are shown as the mean ± SD values. *P* > 0.05 was considered N.S., ***P* < 0.01 and ****P* < 0.001.

**FIGURE S12** Disruption of the EHD1/CD44 interaction promotes Hippo signaling activity and attenuates the stemness of H1299 cells. (A) Flag-tagged full-length EHD1 (EHD1-FL) or EHD1 deletion mutants and Myc-tagged CD44 were co-expressed in H1299 cells. Extracts were subjected to IP with an anti-Myc antibody, and bound EHD1 was analyzed by Western blotting using an anti-Flag antibody. n.s.: non-specific band. (B) Myc-tagged full-length CD44 (CD44-FL) or CD44 deletion mutants and EHD1-Flag were co-expressed in H1299 cells. Extracts were subjected to IP with an anti-Flag antibody, and bound Myc was analyzed by Western blotting using an anti-Myc antibody. n.s.: non-specific band. (C) Western blot analysis showed the effect of disrupting the EHD1/CD44 interaction on the expression of the core components and downstream molecules of the Hippo signaling pathway. (D-E) The effect of disrupting the EHD1/CD44 interaction on the stemness of H1299 cells was indicated by the 3D spheroid cancer models. (F) Holoclone assays showed the effect of disrupting the EHD1/CD44 interaction on stemness. (G) Expression of stemness-related markers in H1299-derived cells. The data are shown as the mean ± SD values. *P* > 0.05 was considered N.S., ***P* < 0.01 and ****P* < 0.001.

**FIGURE S13** Disruption of the EHD1/CD44 interaction inhibits LUAD cells metastasis. (A) Wound healing assays showed the effect of disrupting the EHD1/CD44 interaction on the migration ability and motility of A549 cells. (B) Transwell assays detected the effect of disrupting the EHD1/CD44 interaction on the migration and invasion of A549 cells. (C) Expression of EMT-related markers after disruption of the EHD1/CD44 interaction in A549 cells. (D) Wound healing assays determined the effect of disrupting the EHD1/CD44 interaction on the migration ability and motility of H1299 cells. (E) Transwell assays detected the effect of disrupting the EHD1/CD44 interaction on the migration and invasion of H1299 cells. (F) Expression of EMT-related markers after disruption of the EHD1/CD44 interaction in H1299 cells. The data are shown as the mean ± SD values. *P* > 0.05 was considered N.S., ***P* < 0.01 and ****P* < 0.001.

**FIGURE S14** SP1 depletion inhibits the stemness of LUAD cells. (A) The nine overlapping TFs with the targets of TEAD1 identified using the Cistrome Data Browser and with the upstream targets of SP1 identified using JASPAR and the UCSC Genome Browser. (B-C) The 3D spheroid cancer models showed the effect of SP1 knockdown on the stemness of LUAD cells. Bar graphs showing the quantification of the number of spheres per well formed by LUAD-derived cells. (D) The stemness of LUAD cells with SP1 knockdown was detected by holoclone assays. (E) Expression of stemness-related proteins in LUAD-derived cells after SP1 depletion. The data are shown as the mean ± SD values. *P* > 0.05 was considered N.S., **P* < 0.05, ***P* < 0.01 and ****P* < 0.001.

**FIGURE S15** SP1 knockdown inhibits the metastasis of LUAD cells. (A) Wound healing assays showed the effect of SP1 knockdown on the migration ability and motility of LUAD cells. (B) Transwell assays detected the effect of SP1 silencing on the migration and invasion of LUAD cells. (C) Expression of EMT-related markers after SP1 depletion in LUAD cells. The data are shown as the mean ± SD values. *P* > 0.05 was considered N.S., ***P* < 0.01 and ****P* < 0.001.
