# Supplementary figures and images for: A novel EHD1/CD44/Hippo/SP1 positive feedback loop potentiates stemness and metastasis in lung adenocarcinoma
Source: Clin Transl Med. 2022 Apr 29;12(4):e836. doi: 10.1002/ctm2.836 (PMC9786223; doi:10.1002/ctm2.836)

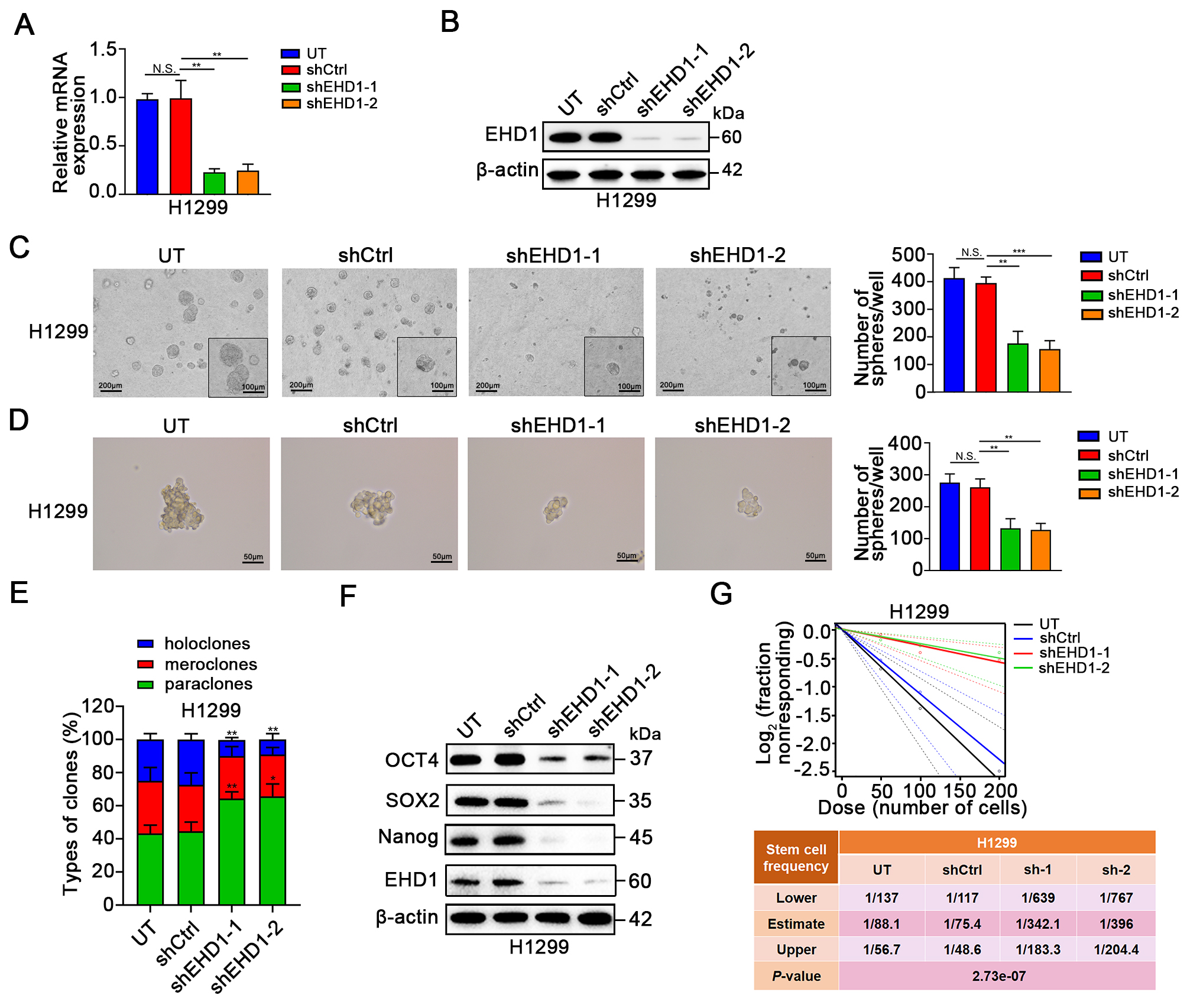

Supplement: Supplementary file 2 — Supporting information [file CTM2-12-e836-s009.jpg]

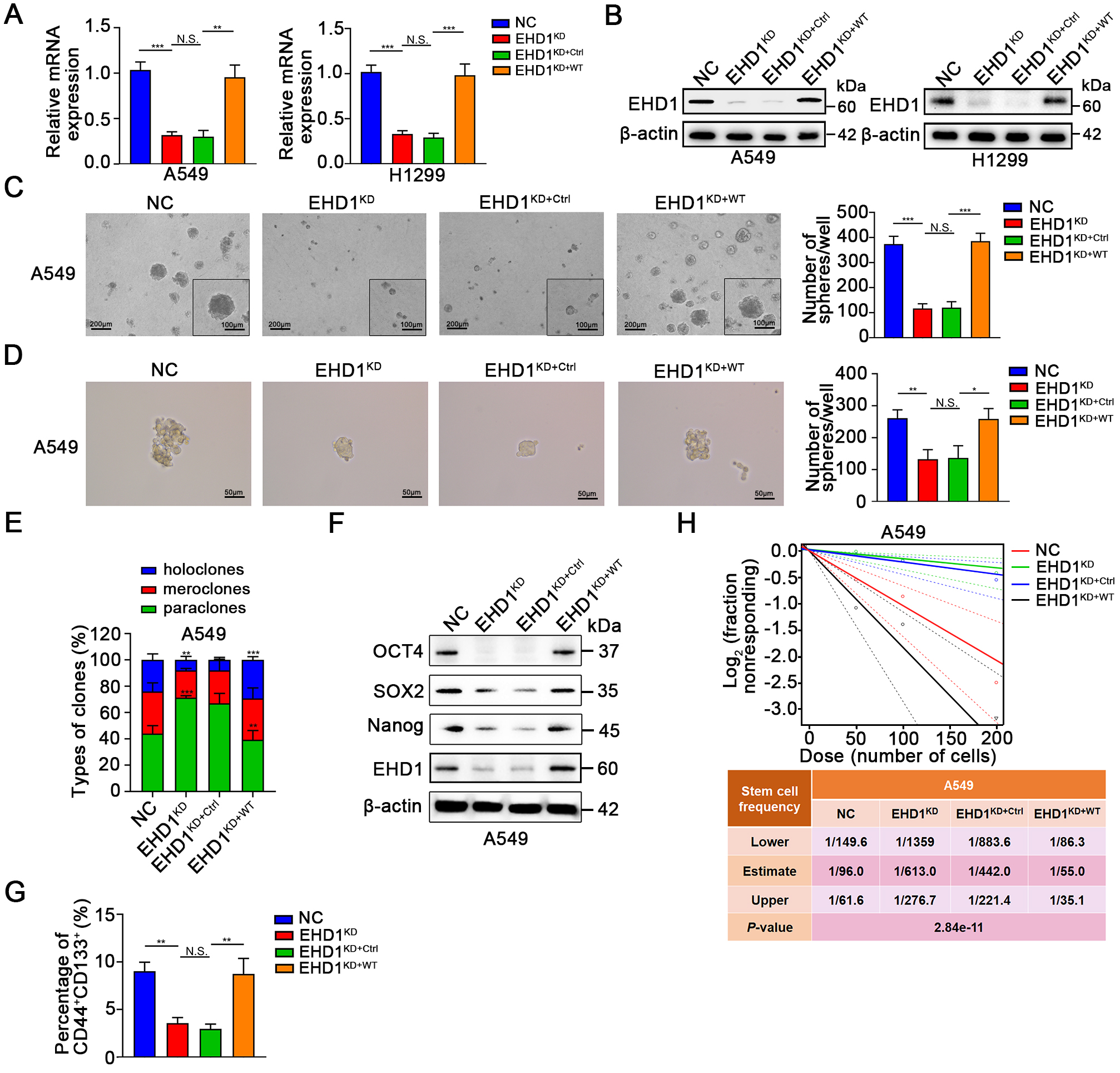

Supplement: Supplementary file 3 — Supporting information [file CTM2-12-e836-s016.jpg]

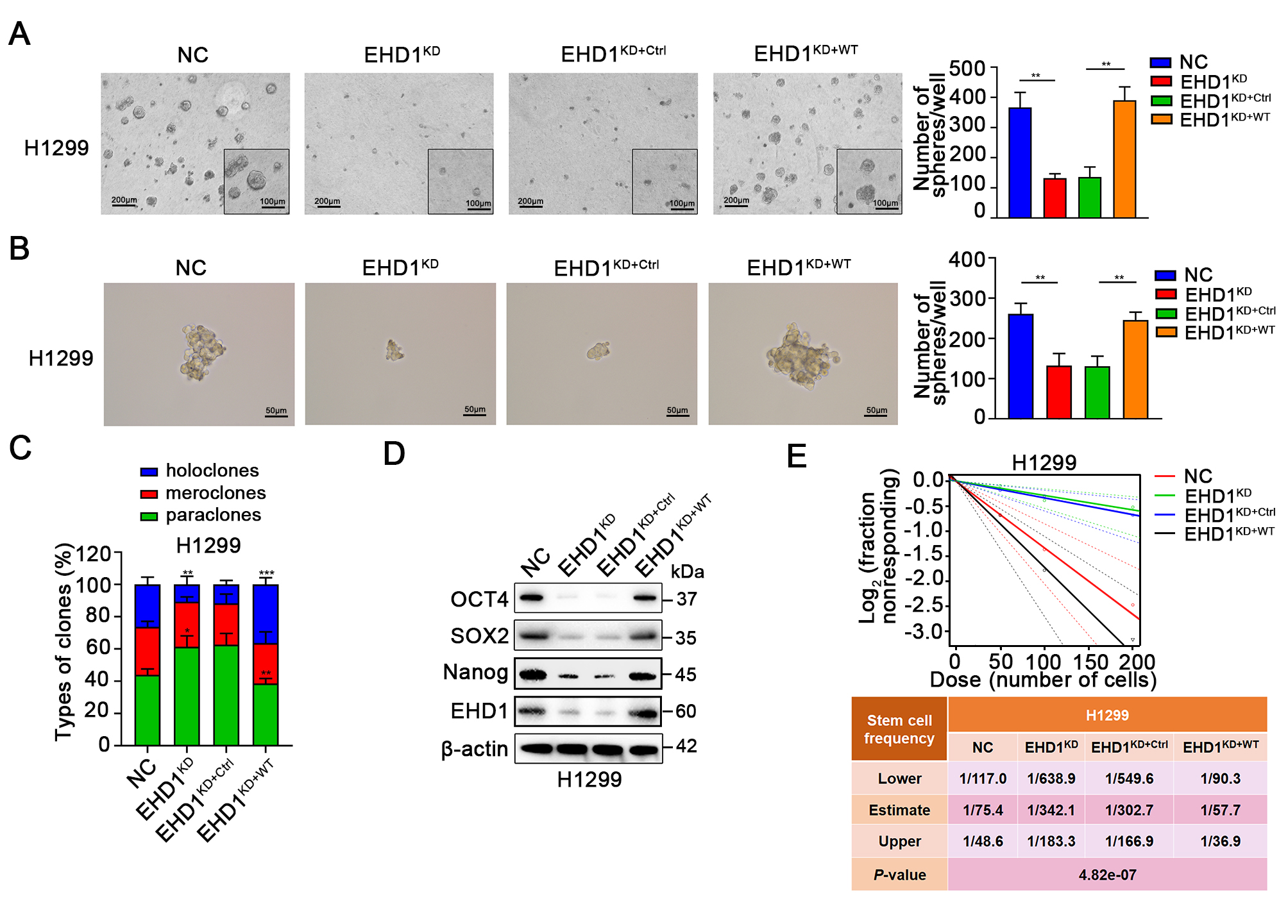

Supplement: Supplementary file 4 — Supporting information [file CTM2-12-e836-s010.jpg]

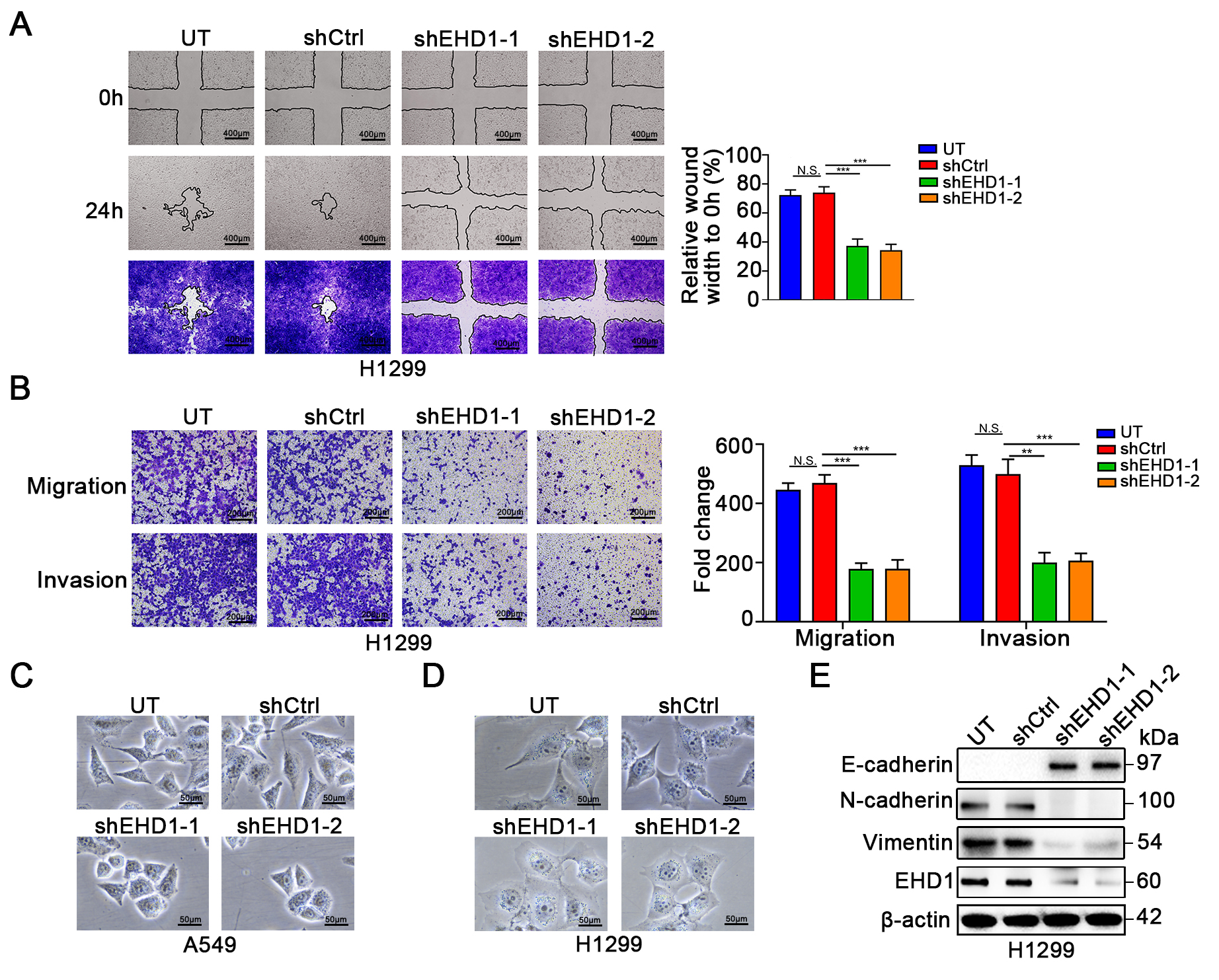

Supplement: Supplementary file 5 — Supporting information [file CTM2-12-e836-s019.jpg]

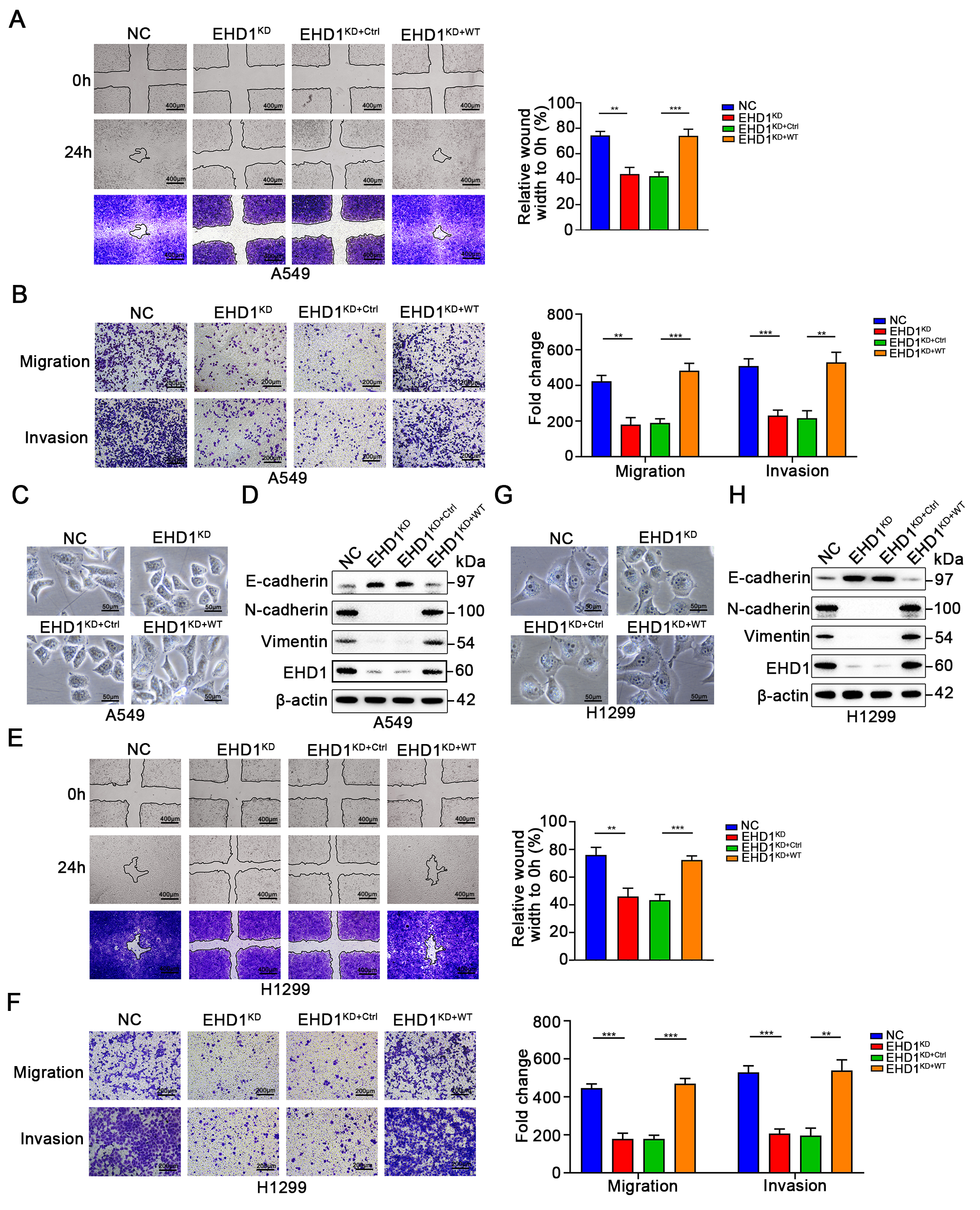

Supplement: Supplementary file 6 — Supporting information [file CTM2-12-e836-s013.jpg]

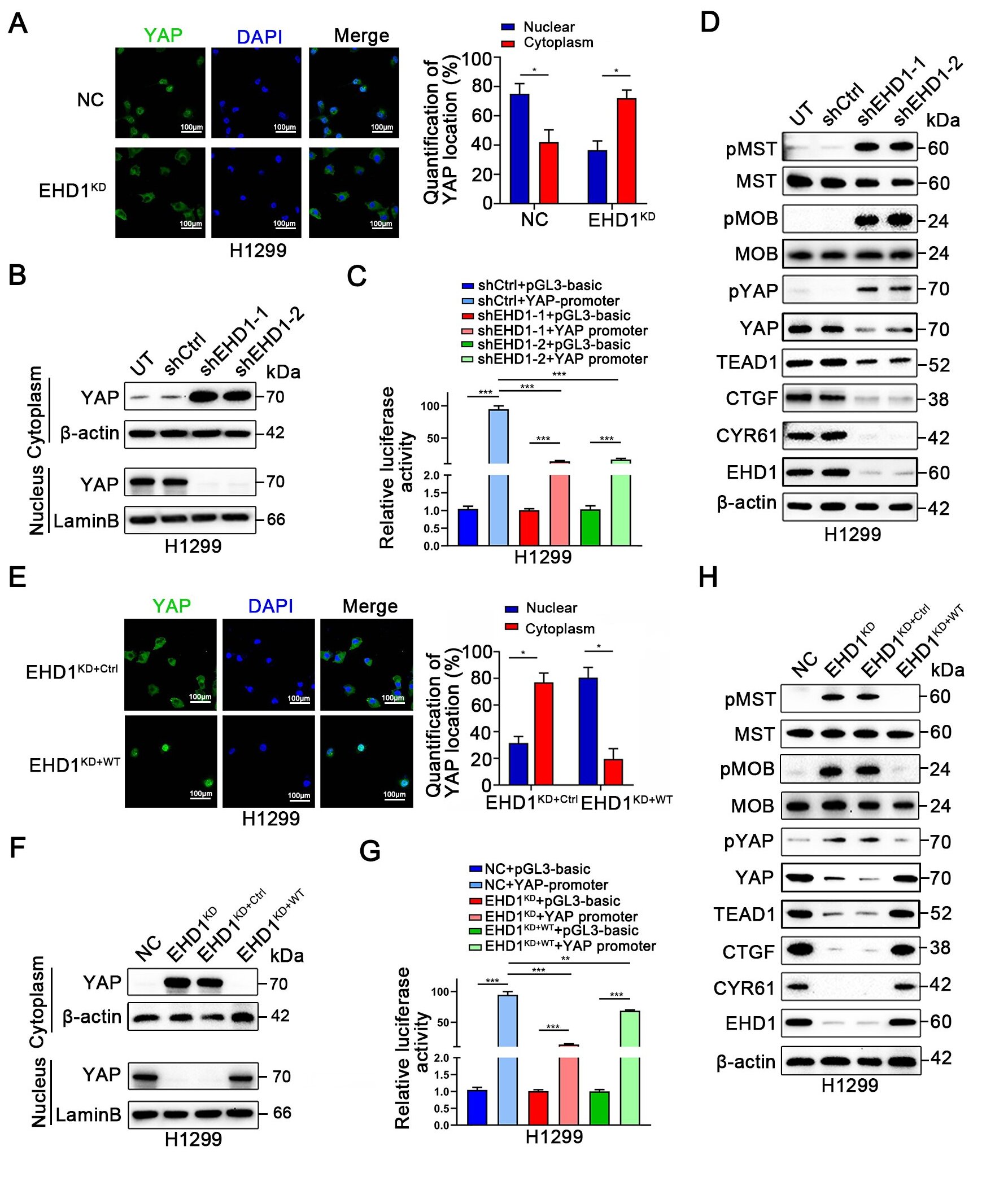

Supplement: Supplementary file 7 — Supporting information [file CTM2-12-e836-s017.jpg]

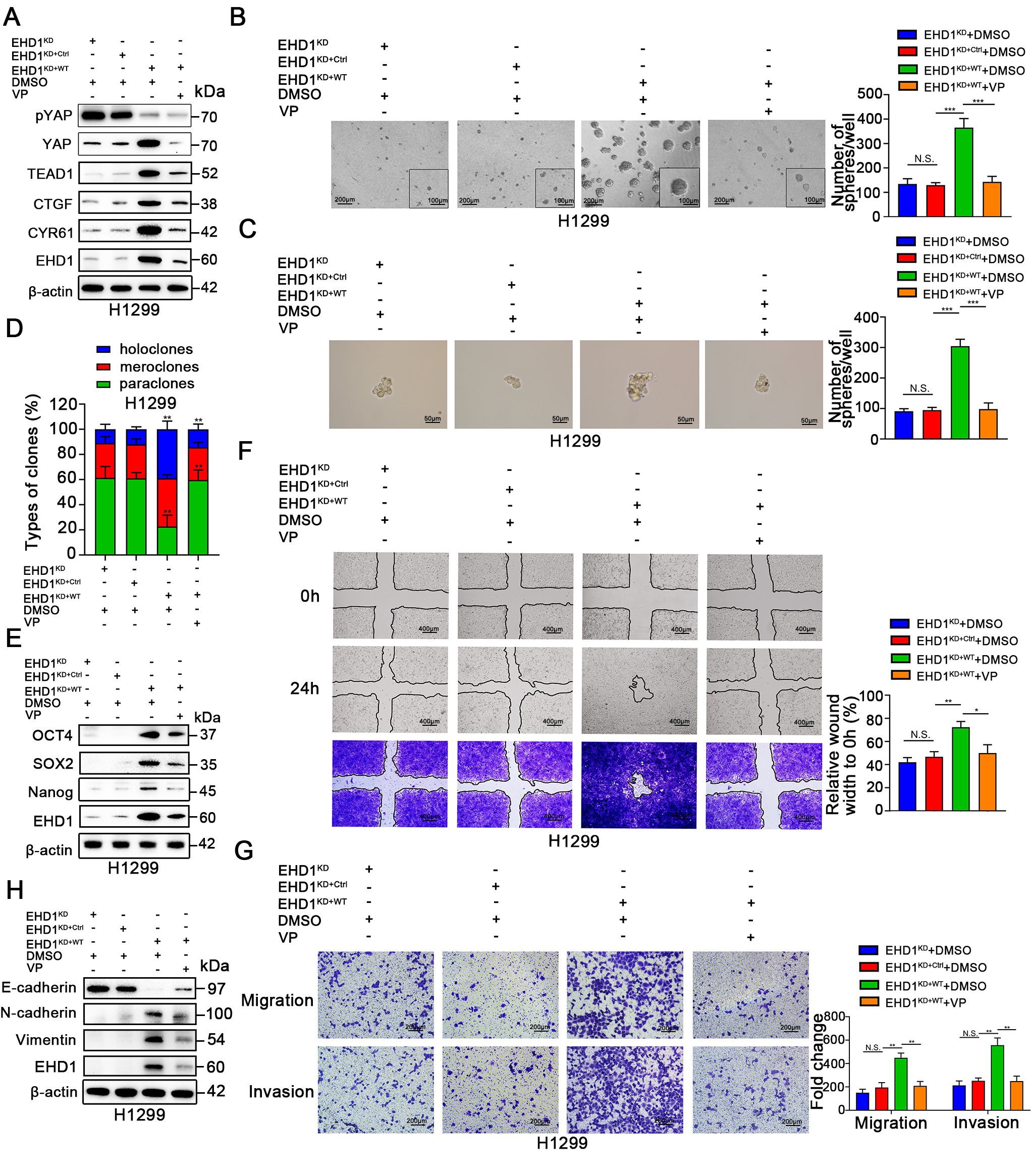

Supplement: Supplementary file 8 — Supporting information [file CTM2-12-e836-s008.jpg]

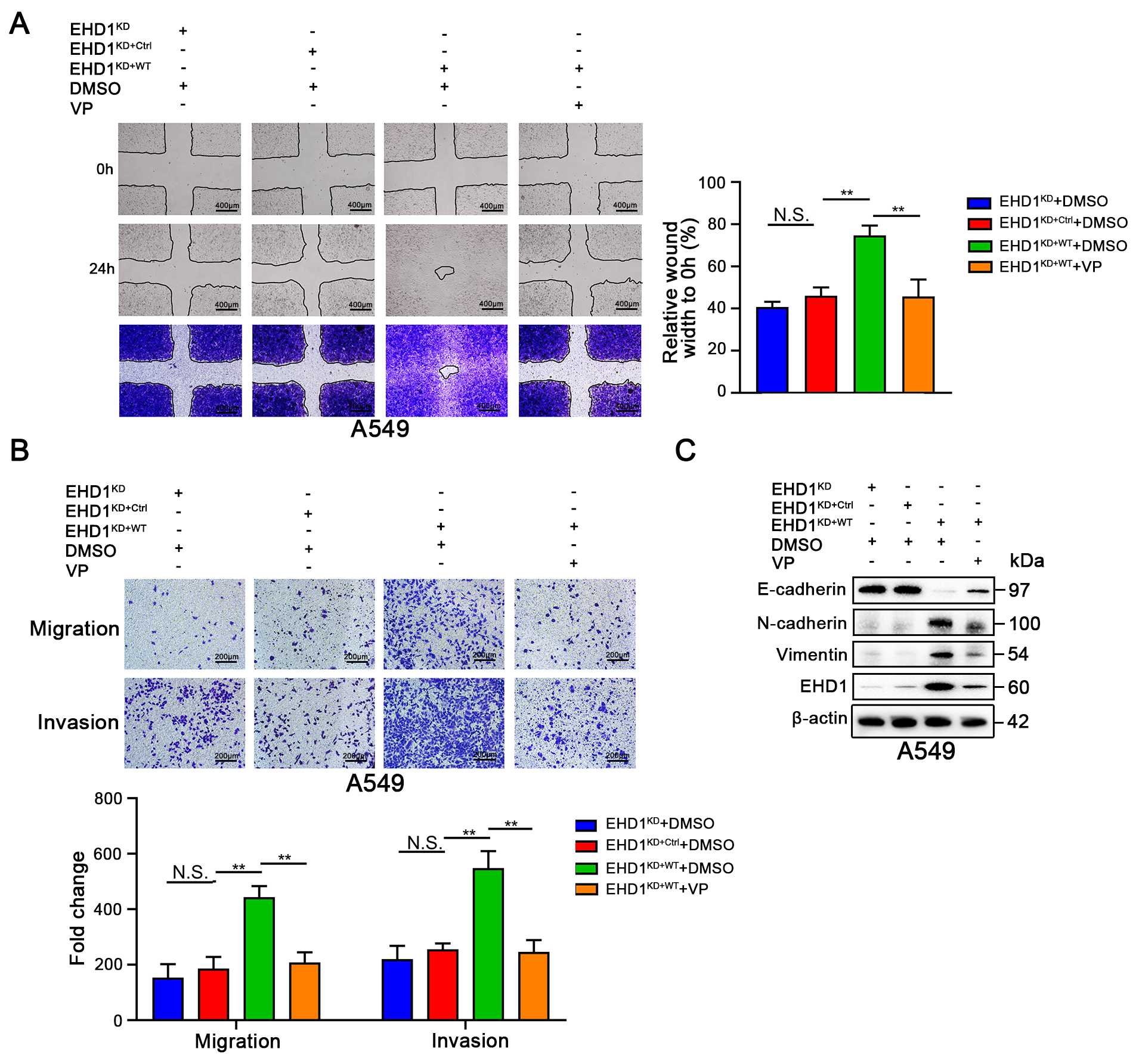

Supplement: Supplementary file 9 — Supporting information [file CTM2-12-e836-s005.jpg]

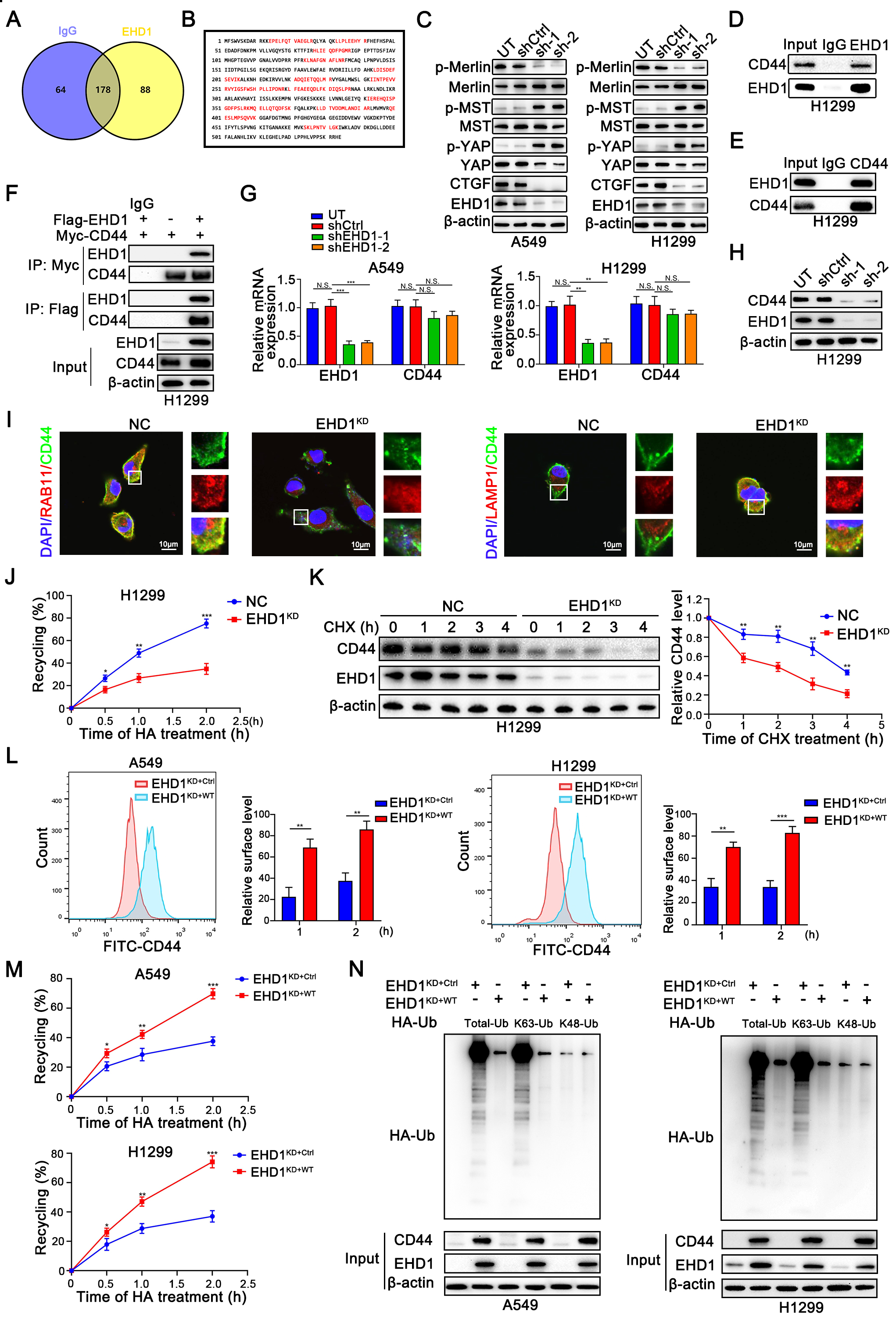

Supplement: Supplementary file 10 — Supporting information [file CTM2-12-e836-s004.jpg]

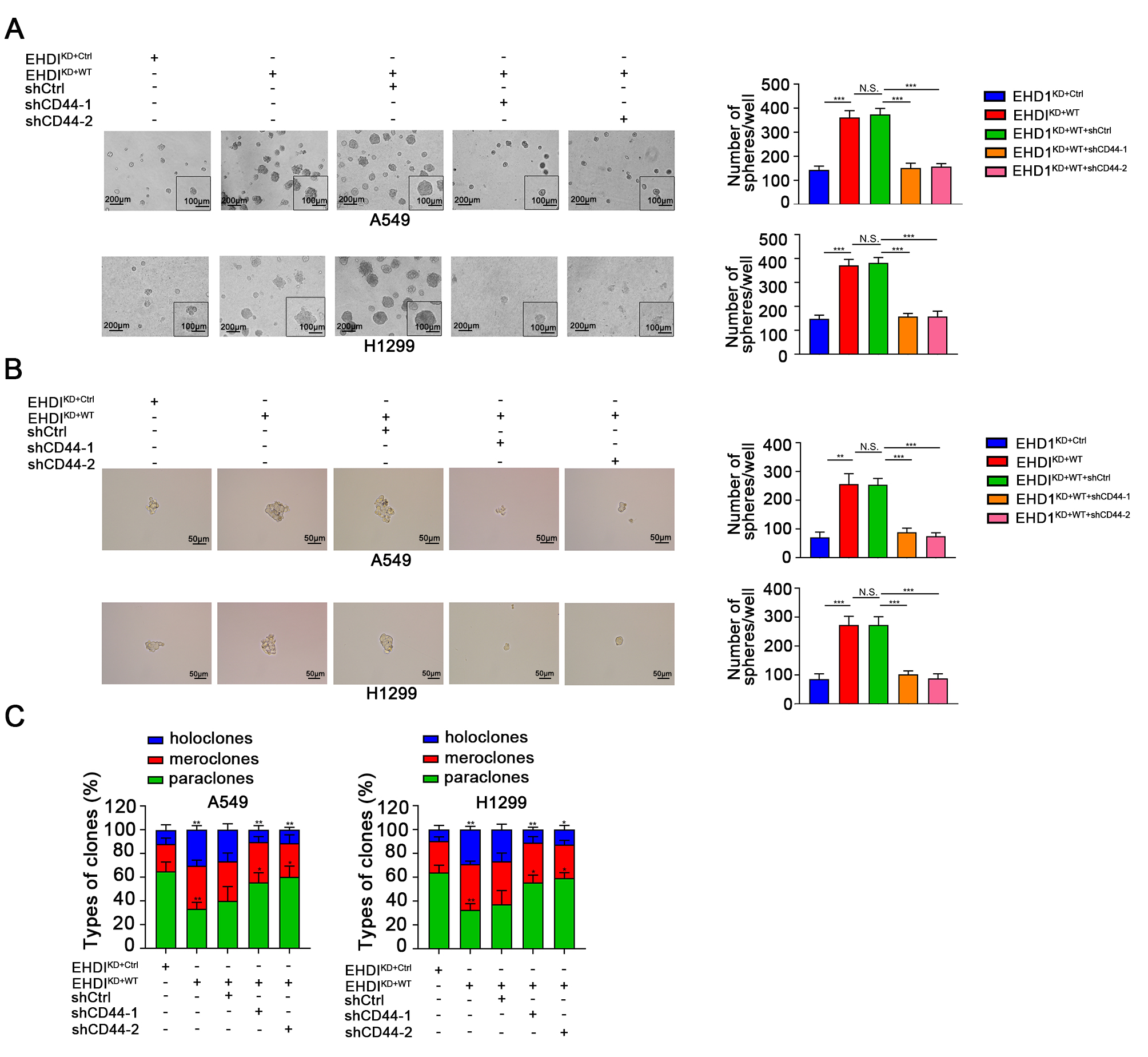

Supplement: Supplementary file 11 — Supporting information [file CTM2-12-e836-s006.jpg]

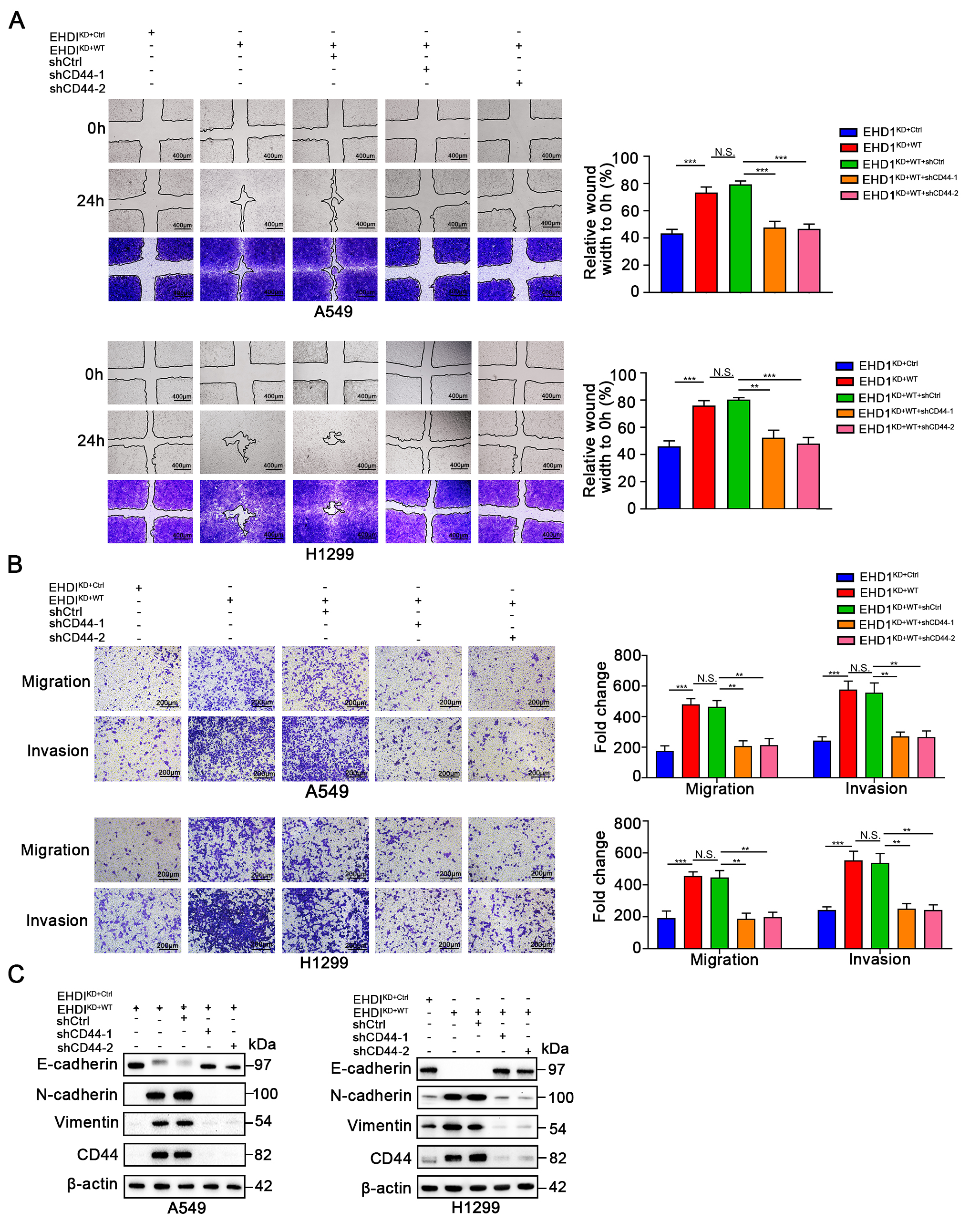

Supplement: Supplementary file 12 — Supporting information [file CTM2-12-e836-s003.jpg]

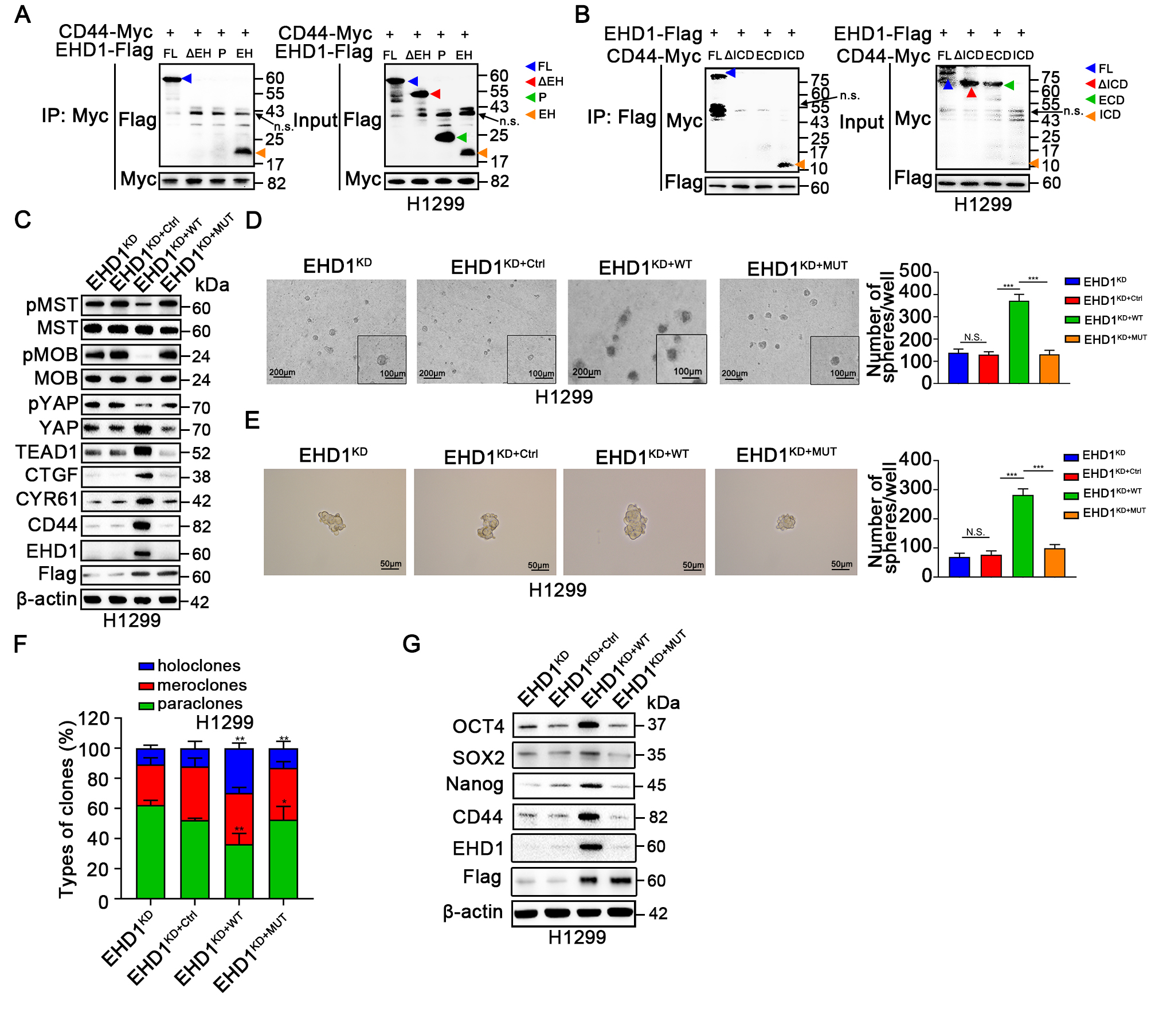

Supplement: Supplementary file 13 — Supporting information [file CTM2-12-e836-s012.jpg]

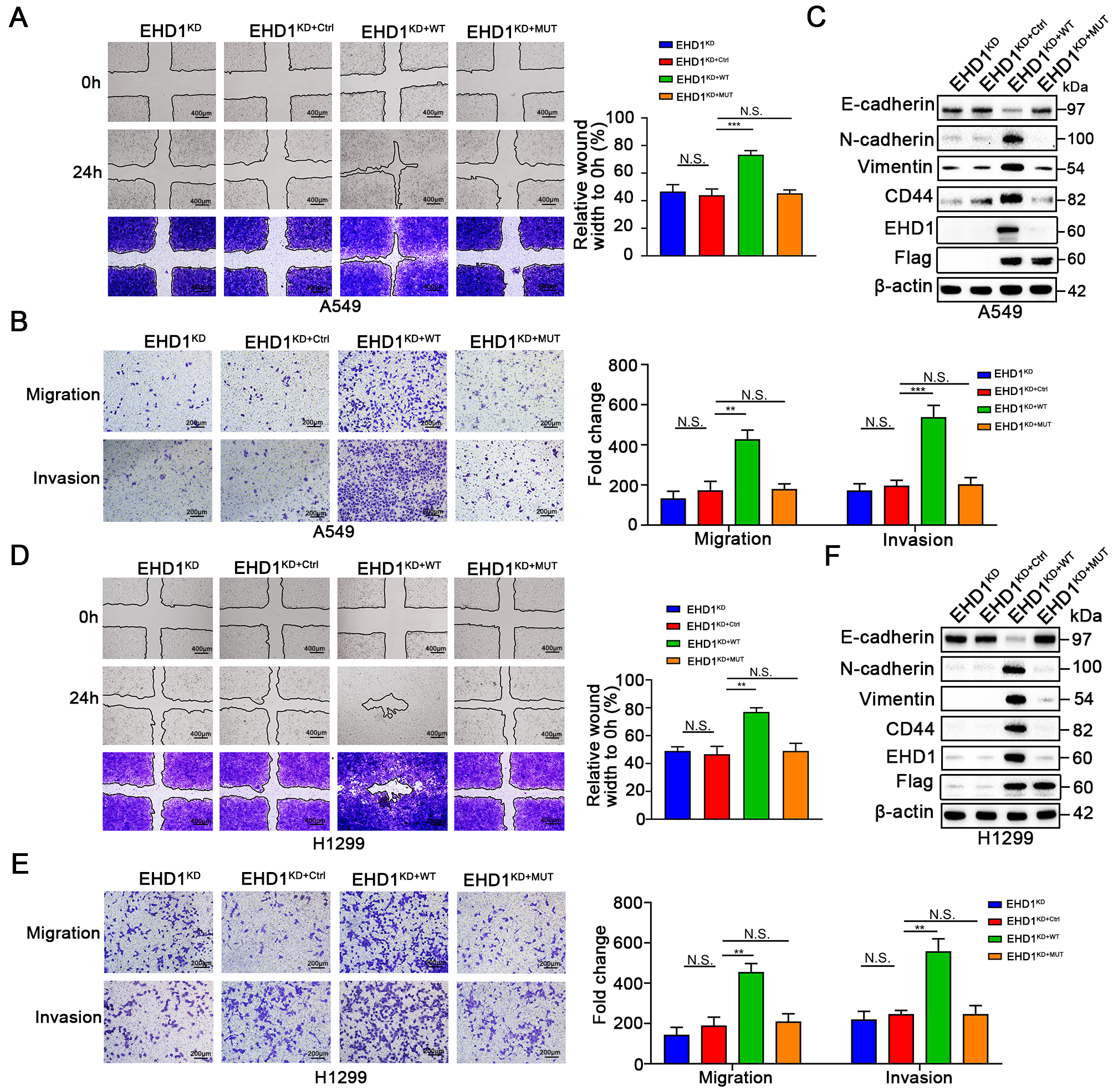

Supplement: Supplementary file 14 — Supporting information [file CTM2-12-e836-s020.jpg]

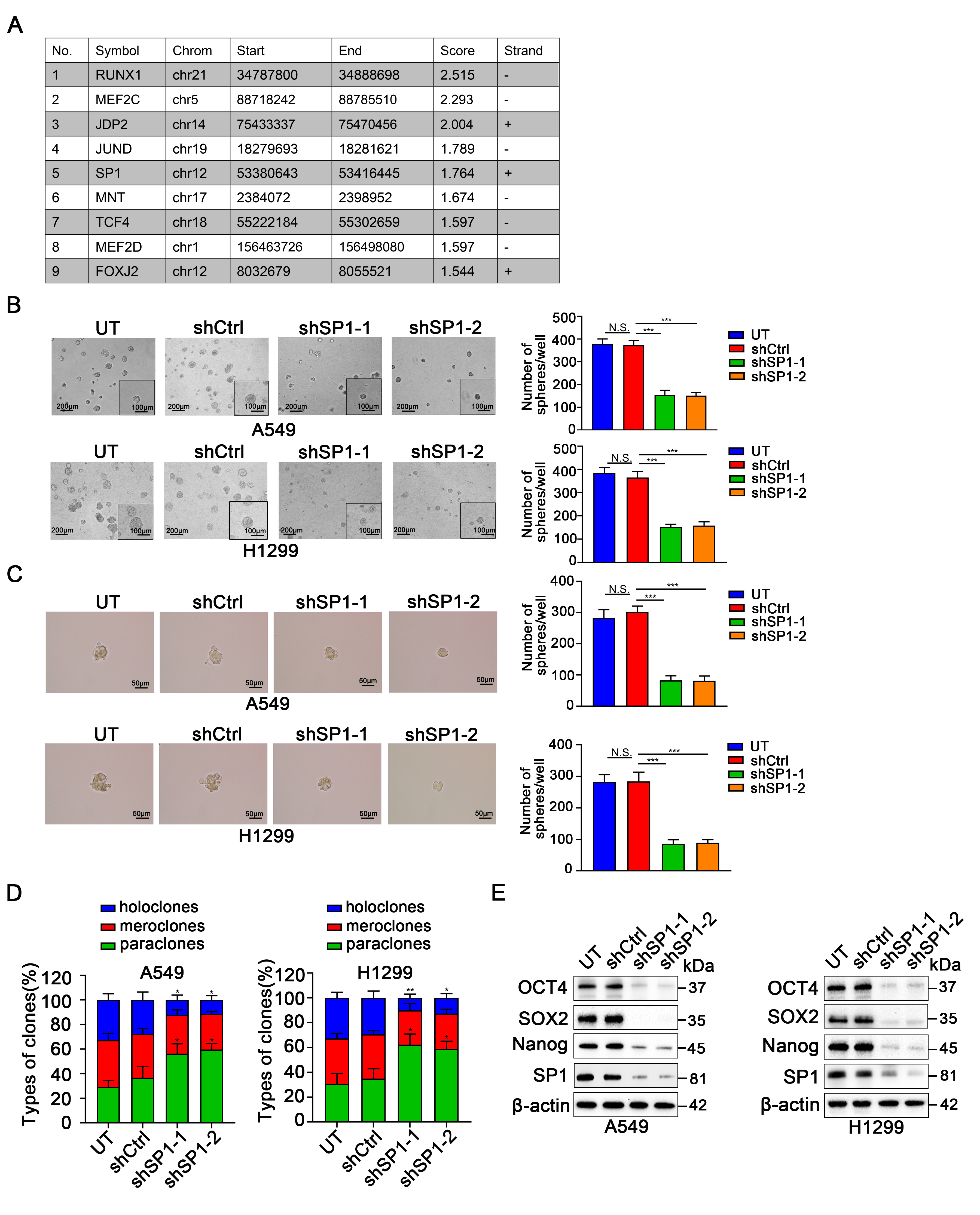

Supplement: Supplementary file 15 — Supporting information [file CTM2-12-e836-s018.jpg]

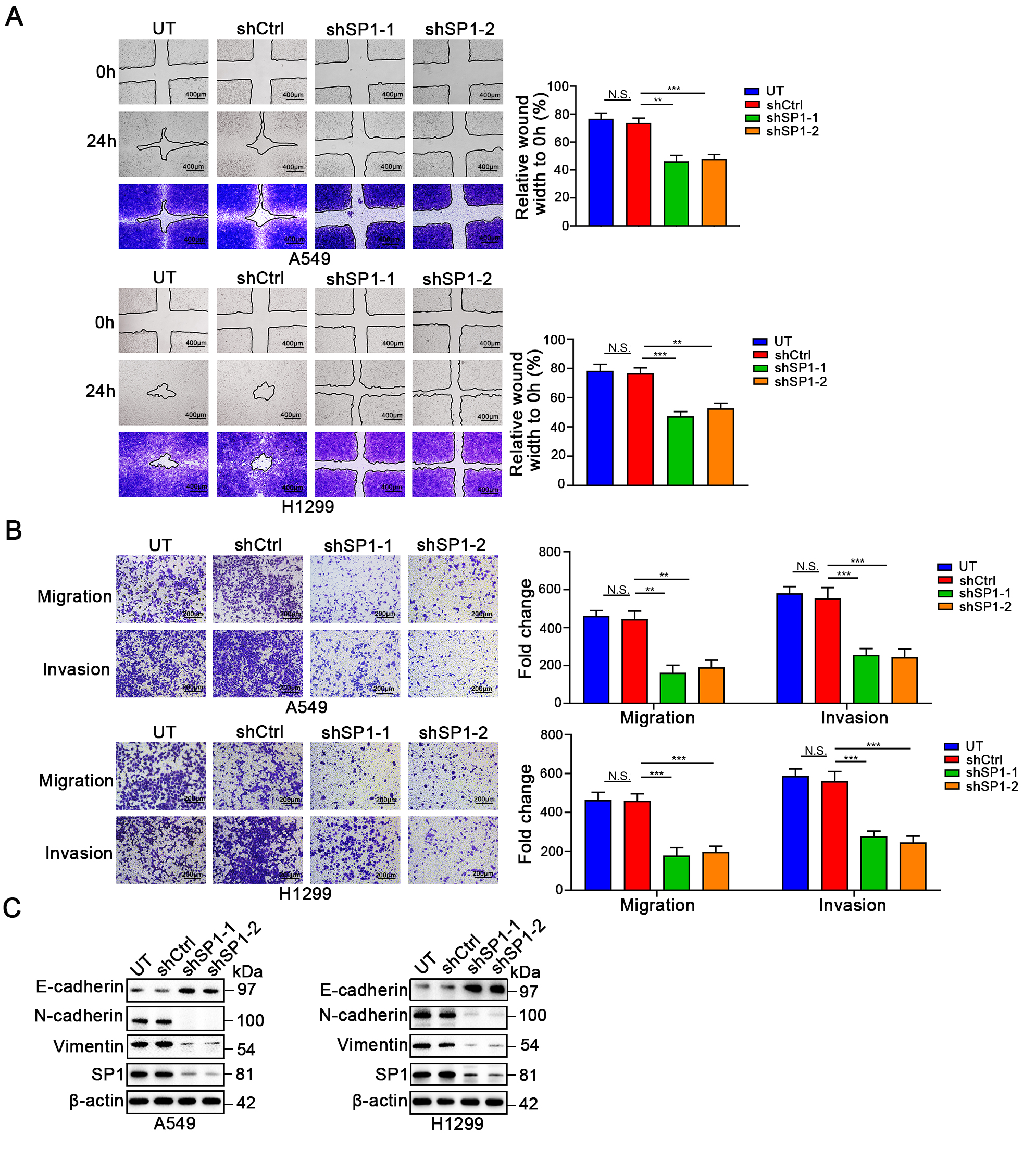

Supplement: Supplementary file 16 — Supporting information [file CTM2-12-e836-s002.jpg]
